# Comorbidity Networks in Cardiovascular Diseases

**DOI:** 10.3389/fphys.2020.01009

**Published:** 2020-08-28

**Authors:** Héctor A. Cruz-Ávila, Maite Vallejo, Mireya Martínez-García, Enrique Hernández-Lemus

**Affiliations:** ^1^Health Promotion Department, Autonomous University of Mexico City, Mexico City, Mexico; ^2^Sociomedical Research Unit, National Institute of Cardiology “Ignacio Chávez”, Mexico City, Mexico; ^3^Computational Genomics Division, National Institute of Genomic Medicine (INMEGEN), Mexico City, Mexico; ^4^Centro de Ciencias de la Complejidad, Universidad Nacional Autónoma de México, Mexico City, Mexico

**Keywords:** comorbidity, cardiovascular disease, biomedical network science, differential diagnostics, genetic conditions

## Abstract

**Background:** Cardiovascular diseases are the leading causes of mortality worldwide. One reason behind this lethality lies in the fact that often cardiovascular illnesses develop into systemic failure due to the multiple connections to organismal metabolism. This in turn is associated with co-morbidities and multimorbidity. The prevalence of coexisting diseases and the relationship between the molecular origins adds to the complexity of the management of cardiovascular diseases and thus requires a profound knowledge of the genetic interaction of diseases.

**Objective:** In order to develop a deeper understanding of this phenomenon, we examined the patterns of comorbidity as well as their genetic interaction of the diseases (or the lack of evidence of it) in a large set of cases diagnosed with cardiovascular conditions at the national reference hospital for cardiovascular diseases in Mexico.

**Methods:** We performed a cross-sectional study of the National Institute of Cardiology. Socioeconomic information, principal diagnosis that led to the hospitalization and other conditions identified by an ICD-10 code were obtained for 34,099 discharged cases. With this information a cardiovascular comorbidity networks were built both for the full database and for ten 10-years age brackets. The associated cardiovascular comorbidities modules were found. Data mining was performed in the comprehensive ClinVar database with the disease names (as extracted from ICD-10 codes) to establish (when possible) connections between the genetic associations of the genetic interaction of diseases. The rationale is that some comorbidities may have a stronger genetic origin, whereas for others, the environment and other factors may be stronger.

**Results:** We found that comorbidity networks are highly centralized in prevalent diseases, such as cardiac arrhythmias, heart failure, chronic kidney disease, hypertension, and ischemic diseases. Said comorbidity networks are actually modular on their connectivity. Modules recapitulate physiopathological commonalities, e.g., ischemic diseases clustering together. This is also the case of chronic systemic diseases, of congenital malformations and others. The genetic and environmental commonalities behind some of the relations in these modules were also found by resorting to clinical genetics databases and functional pathway enrichment studies.

**Conclusions:** This methodology, hence may allow the clinician to look up for non-evident comorbidities whose knowledge will lead to improve therapeutically designs. By continued and consistent analysis of these types of patterns, we envisaged that it may be possible to acquire, strong clinical and basic insights that may further our advance toward a better understanding of cardiovascular diseases as a whole. Hopefully these may in turn lead to further development of better, integrated therapeutic strategies.

## 1. Introduction

Cardiovascular diseases are the leading cause of human mortality worldwide (Mittal et al., 2019). Most of these deaths are related to aging and often coexist with other diseases affecting the individual's function and survival. To account for this, the term *comorbidity* was coined to represent the occurrence of other medical conditions in addition to an index condition of interest (Ng et al., 2012). Such comorbidity relationships occur whenever two or more diseases are present in the same individual more often than by chance alone (Faner et al., 2014). Recent times have witnessed dramatic advances in medical research characterized by moving forward from the single-disease focused model to a systemic, patient-centered view, such approaches progressively permeate into medical technology, and therapeutics resulting in significant reduction in morbidity and mortality in some specific cases. However, less attention has been paid to the design of clinical guidelines that are still mostly devoted to treating single maladies (Bell and Saraf, 2016).

A relevant issue is then how do comorbidities associate among them (comorbidity clustering), because of the implications of this phenomenon in terms of disease-disease and drug-drug interactions. In fact, if the therapy recommended for one disease is contraindicated in the presence of a concurrent medical condition, the usefulness of clinical practice guidelines becomes limited. In such a scenario, evidence-based treatment guidelines, designed for single diseases, can lead to serious therapeutic conflicts (Maggi et al., 2019). To circumvent such limitations, a personalized approach to medicine may benefit from the inclusion of ideas from a somewhat recent field of research, generally known as *systems biology* or *network medicine*—when applied to humans. This approach offers the potential to decipher and understand the relationships between comorbidities or multimorbidities at a much deeper level by considering coordinated instances (systems) rather than single conditions (Faner et al., 2014).

Within this theoretical framework, the concept of diseasome was introduced to indicate that although often treated separately, most human diseases are indeed interdependent. These ideas lead to the construction of the so-called human disease network (HDN), a graph in which two diseases are connected if they have a common genetic, regulatory or metabolic origin and/or common protein protein interactions. Analyzing HDN lead to the identification of disease modules as well as systematic finding of druggable pathways, and to a *cartography* of the molecular relationships behind multimorbidity (Faner et al., 2014; Sun et al., 2014). In the following section, we will provide a description of how we address these ideas in this work.

## 2. Materials and Methods

Since the inception of HDNs, a number of different approaches have been developed, there are, in general, three types of disease-linking networks, based on three different formalisms: diseases are linked based on shared genes; disease connections reflecting shared metabolic pathways; and disease comorbidity networks, where links between diseases are based on their significant co-occurrence. In this work we will start by considering a comorbidity network, and then we will analyze the genetic and functional relationships *within this network*.

**Figure 1:**
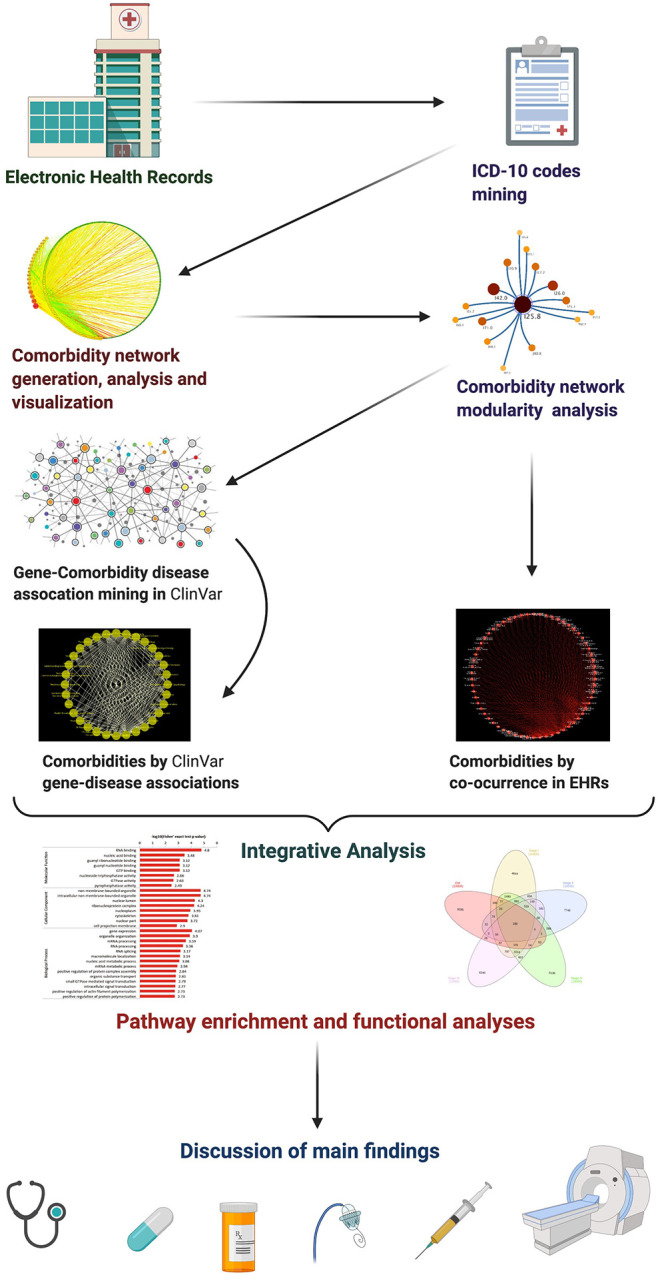
Schematic representation of the diverse layers of the methodology applied in this article.

We will thus analyze an empirically based cardiovascular comorbidity network, curated from clinical data at the national reference hospital for CVDs in Mexico. From this network, cardiovascular comorbidity modules were inferred, hand-curated mapping of disorder gene associations and cardiovascular comorbidities Jaccard coefficient analyzed, followed by discussion of the results. We used as biological data annotations from ClinVar. Figure 1 shows a schematic representation of the methodology applied in this article.

### 2.1. Data Acquisition (Electronic Health Records)

The National Institute of Cardiology “Ignacio Chávez” (NIC-ICh) is the referral hospital for specialized cardiovascular care in Mexico (Vargas-Alarcón et al., 2010). The NIC-ICh is a public hospital for specialized cardiovascular care, since 1944 it was the first of its kind in the world. It has been a hospital center for the care of the patient with few resources who lacks social security; a large research laboratory (of basic science and applied clinical type); as well as a graduate school where specialists in cardiology and related branches are trained. The NIC-ICh is the flagship specialized institution for the treatment of cardiovascular diseases in Mexico, it is also a third level hospital receiving in-patients with related ailments, such as metabolic, inflammatory, and systemic diseases, whose treatment may involve immunology, rheumatology, nephrology, and similar ailments in addition to *pure* cardiology-related treatments.

We used the Electronic Health Record (EHR) Database entries between January 1, 2011 and December 31, 2016. This database contains the socioeconomic information and the principal diagnosis led to hospitalization, as well as other diseases, disorders, conditions or health problems. International Classification of Diseases, tenth revision (ICD-10) was used to identify and classificate them, by an ICD-10 code. The EHR management procedures of the Institution are set to provide up to five main comorbidities. In order to minimize biases we have considered as our population, the full set of hospital discharged patients in the time period under study, with the exception of those with incomplete or erroneous coding. To be clear, all types of diagnostics present in the EHRs are considered (see inclusion and exclusion criteria below). The study population included 34,099 discharged cases. The cardiovascular comorbidities assessed included any disease registered in each case.

Inclusion and exclusion criteria were as follows:

- Inclusion criteria
  1. Clinical records of any geographic region.
  2. Clinical records of any socioeconomic level.
  3. Clinical records of any sex.
  4. Clinical records of any age.
  5. Clinical records of any medical or hospital service.
  6. Clinical records of any comorbidities.
  7. Clinical records of any cause of death.
- Exclusion criteria
  1. Clinical records with incomplete information.
  2. Clinical records with non-existing codes.

### 2.2. Data Processing (Coding)

The EHR data was processed using custom code (in the R programming language) for the design and analysis of a network of comorbidities, which would then be used to infer communities of comorbidities, which eventually lead to a connection between genes and their metabolic pathways for each community group studied. Programming code for this study is available in the following public access repository: https://github.com/CSB-IG/Comorbidity_Networks.

Information of comorbidities or coexisting illnesses was based on diagnoses and procedures mapped to ICD-10 codes, and genes associated with each disease were searched and coded according to the ClinVar database. ClinVar is a large public archive of reports that collects information on genomic variants and their relationships with human health (Landrum et al., 2017; Landrum and Kattman, 2018).

ClinVar has become a valuable resource to support clinical variant interpretation and provides a growing resource for studying genotype and phenotype correlations. ClinVar contains 503,065 unique genetic variants from 1,229 submitters from all around the world. ClinVar provides for each variant entry more than 30 fields of data that come in multiple levels and are connected to external resources, for example, the National Center for Biotechnology Information (NCBI), the NCBI database of genetic variation (dbSNP), PubMed Central or the Reference Sequence Database (Pérez-Palma et al., 2019).

Some of the disease names or ICD-10 codes obtained from the EHR database and those used in the ClinVar database by geneticists are not identical. Therefore, we map manually, but very carefully the ClinVar disease names into ICD-10 codes and established connections between the genetic associations and the comorbidity measures in order to obtain a dataset for the experimental models. Those incorrectly registered disease names or codes were discarded from the beginning of data curation.

It is worth noticing that the use of ICD-10 codes in research presents a number of challenges and limitations, since the system was originally developed with hospital administration and cost-estimation purposes, rather than as a controlled vocabulary for standardized clinical reporting.

It is known that ICD codes were introduced as a support for hospital administrative databases, which are often aimed to obtain reimbursement. They were not explicitly planned for clinical research. With this in mind, when these codes are used for clinical purposes, it is necessary to carefully evaluate them, since the actual subjects of interest may not be accurately defined. This may be critical in the assessment of chronic conditions. Moreover, ICD codes perform better with sets of diseases enriched for frequent, well-known conditions. A broad discussion on the scope and limitations of ICD-10 coding, as used in this work, is presented as “Supplementary Document 1.”

Regarding the specific case of EHRs in the NIC-ICh, it is worth noticing that its administrative database coding, archiving and retrieval procedures have been certified and validated by the World Health Organization by means of the local “Collaborating Center for WHO International Classification Schemes—Mexico Chapter” (CEMECE, for its Spanish acronym). These procedures are in agreement with ISO 9001:2000, ISO/IEC 27001 certifications and with the Official Mexican Norm (NOM for its Spanish acronym): NOM-004-SSA3-2012.

### 2.3. Cardiovascular Comorbidity Network (CVCnetwork)

Once the mining of the medical cases was carried out, an *undirected network*, was built based on the significant co-occurrent diseases coded according to ICD-10. The *node* is the unit of analysis of the main disease network represented on a *connectivity map*. Since this work is founded in the framework of the theory of complex networks, some of the *technical terms* may not be familiar to readers in the clinical research setting. An introductory briefing, to provide for the necessary concepts and terminology has been included as “Supplementary Document 2.”

The origin and destination nodes are diseases that are identified with a default code of ICD-10. Subsequently, a link was drawn between these nodes, as long as at least two diseases occurred in the same person more often than by chance alone [hypergeometric test, with a False Discovery Rate (FDR) multiple testing correction *FDR* < 0.05].

In network theory, one of the parameters used to evaluate the connections in the graph is the *degree centrality* (DC), the total number of links on a node or the sum of the frequencies of the interactions. The degree distribution of a disease is the number of ICD-10 codes associated with that disease. The “Network Analyzer” plugin (Doncheva et al., 2012) in the “Cytoscape” open source network analysis suite was used to explore and visualize the network (Kohl et al., 2011).

### 2.4. Cardiovascular Comorbidities Modularity (CVCmodules)

*Modularity* or *clustering* is a property that allows further analysis of *local structural properties of a network* that lead to the appearance of subunits known as strongly interconnected *modules* or *communities* (Alcalá-Corona et al., 2018b); that is, it contains a greater number of links between nodes within the community, than the number of links to nodes outside it (Alcalá-Corona et al., 2018a).

Recently, network modularity studies have been used to unveil clinically relevant comorbidity patterns (Barabási et al., 2011; Divo et al., 2015; Choi et al., 2017; Guo et al., 2019). In a similar way to cluster analysis is to assign diseases into modules or communities, so that nodes in the same community are strongly associated with one another than entities from different clusters. The accurate use of *community detection analysis* of comorbidity networks to identify comorbidity patterns depending upon how the coincidental comorbidity is accounted for Ng et al. (2012).

A community grouping algorithm—based on *random walks and information theory* focused on the interdependence of the links—called *Infomap* (Rosvall and Bergstrom, 2008) was used to infer the modules in the comorbidity network. For the modular visualization of these subunits the online application called *MapEquation* (Bohlin et al., 2014) was used. Modules were labeled with the name of the node, of the ICD-10 code for the disease, with the highest *PageRank* index (PRI) on it (Brin and Page, 1998). This is a *measure of centrality* based on various algorithms used to numerically assign the relevance of the main diseases, in this way, communities constitute the units of structured diseases of greater complexity than individual diseases (Rosvall and Bergstrom, 2008).

### 2.5. Cardiovascular Comorbidities-Gene Associations (CVCgenes)

From the obtained modules, the three largest were selected. A semi-supervised curation of coincidences between associated genes in the “ClinVar” database was performed for these modules using a custom-made comparison program. ClinVar is a freely accessible, public archive of reports of the relationships among human genetic variations and phenotypes, with supporting evidence from the published literature, clinical trials and other accountable reports. With the information obtained, tables were made with variables that contain, on the one hand, the codes of the diseases compared and the other number of gene matches found.

The *Jaccard index* was used to measure the degree of genetic similarity between the two diseases. The Jaccard Index (JI) is a reliable measure of similarity between two sets, being the ratio of the size of the intersection of the sets to the size of their union, for two sets A and B this is written as:

(1) $$\begin{array}{l} JI_{A,B}=\frac{n\left(A⋂B\right)}{n\left(A⋃B\right)} \end{array}$$

This way, two completely different sets will have *JI*_*A,B*_ = 0, whereas two identical sets will have *JI*_*A,B*_ = 1.

### 2.6. Cardiovascular Comorbidities Pathway Enrichment (CVCpathways)

From a semi-causal or mechanistic point of view, comorbidities may arise due to a common genetic background, due to shared environmental and risk factors, or, more likely due to a combination of both with a broad range of proportions from the former and the latter. In order to analyze the extent of the genetic contribution in the chosen examples we have decided to implement *pathway enrichment analysis* (García-Campos et al., 2015) in the lists of shared genes for each comorbidity pair.

Pathway enrichment analysis for a given list of genes involves the search for annotations of biological functions for all the different genes in biological databases, such as KEGG and Gene Ontology. A gene set is said to be *significantly enriched* for a given function if there are more genes annotated as related to that function than would be found by chance alone and then performing a statistical significance test on the size of this difference. In this case, hypergeometric tests with false discovery rate (FDR) corrections for multiple testing were applied. An FDR-corrected hypergeometric test was performed on each list with a significance threshold corrected *p*-value <0.05, using the “Webgestalt” online tool (Liao et al., 2019).

### 2.7. Age-Grouped Cardiovascular Comorbidity Networks (ACVCnetworks)

Clinical comorbidities are differentially present among the diverse age groups. For instance, congenital diseases are more common in newborn, infants and children, whereas chronic degenerative diseases often occur in later stages in life. In order to capture to what extent comorbidities in cardiovascular diseases vary along different age groups, we have stratified our patient database in 10 non-overlapping age brackets (spaced by 10-years differences) ranging from newborn to centennial and we have built their age-tagged comorbidity networks, following the same methodological principles just presented in the previous subsection.

## 3. Results

### 3.1. General Features

We used the EHR Database of the National Institute of Cardiology “Ignacio Chávez” to deploy the study population. Principal diagnosis led to the hospitalization by sex and by age 34,099 discharged cases with cardiovascular diseases were included. They were extracted from electronic medical records (including the International Classification of Diseases code version 10 [ICD-10]) at discharge. We defined comorbidity as the concurrent presence of ≥2 chronic diseases. Comorbidities were summed to provide a dataset of the number of comorbidities in the entire cohort and stratified according to age and sex. The most prevalent comorbidities, in both male and female were related to ischemic heart disease, congenital malformations of heart and kidney diseases.

With regard to women, we can see that *Congenital malformations of the circulatory system* **(Q20–Q28)**, are the most representative, with the highest incidence percentage (94.60%), in the age range of 0–10 years and the incidence of the disease decreases with increasing age. Regarding *Chronic rheumatic heart diseases* **(I05–I09)**, these are manifested most frequently between the 40 and 70 years old (y.o.). For *Ischemic heart disease* **(I20–I25)**, the highest incidence is seen beginning in the 50 y.o. and increases in the following decades until the 80s. And *Other forms of heart disease* **(I30–I52)**, mostly diagnosed in women of age 91–100 y.o. (54.64%), is present throughout all the years.

On the other hand, the group of men is also affected by *Congenital malformations of the circulatory system* **(Q20–Q28)**, in ages 0–10 y.o. (93.07%). Regarding *Chronic rheumatic heart diseases* **(I05–I09)**, these are manifested much less in men, on the contrary, *Ischemic heart diseases* **(I20–I25)** are noticeably manifested more frequently in men than in women from the age of 40–80 y.o. In both groups there was no difference regarding the *Diseases of the genitourinary system*, but *Renal failure* **(N17–N19)** were the most incidents in almost all ages. These general results will be further discussed when we present the age grouped cardiovascular comorbidity networks (ACVCnetworks).

### 3.2. A Cardiovascular Comorbidity Network (CVCnetwork)

We modeled the cardiovascular comorbidity data as a *graph*, the cardiovascular comorbidity network (CVCnetwok): nodes represent the codes of diseases (ICD-10), and *undirected edges* between a pair of coexisting diseases (see Methods). The CVCnetwork constitutes a *single component connected*, all diseases are directly or indirectly connected. The number of nodes is: 1,473, and the number of edges: 20,543.

Despite the large number of connections, this is a relatively low density network (network density = 0.019). This means that of all possible comorbidity relations between the 1,473 diseases, only about 2% of them are actually found in our corpus. By further considering that some diseases (such as arrhythmias, heart failure, and chronic kidney disease, as we will see below) have hundreds of reported comorbidities, this means that multimorbidity is quite heterogeneous, with a few highly multimorbid conditions (such as the ones mentioned above) and many others with few comorbidities.

**Figure 2:**
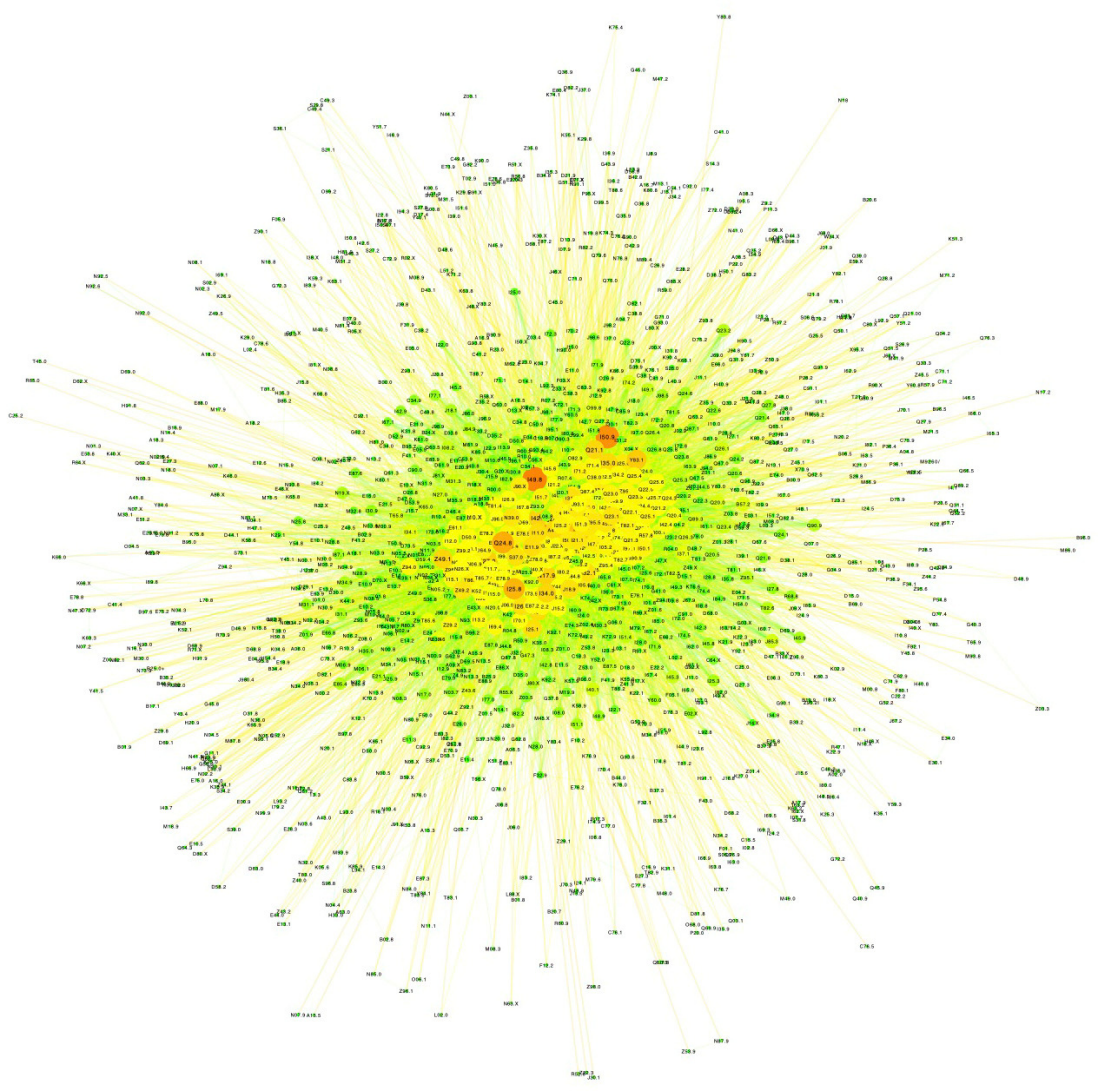
Main comorbidity network. Nodes correspond to the most common connected comorbidities. Node size and color are proportional to the connectivity degree. Small, reddish nodes on the periphery are, the less connected diseases, whereas bigger green nodes at the core are highly connected diseases.

Diseases with the highest number of connections are: *Other specified cardiac arrhythmias* **(I49.8)** with 707 disease neighbors; *Heart failure, unspecified* **(I50.9)** with 629; *Chronic kidney disease, unspecified* **(N18.9)** with 626 disease neighbors; then, *Essential (primary) hypertension* **(I10.X)** with 564; *Other specified congenital malformations of heart* **(Q24.8)** with 490, *Other forms of chronic ischemic heart disease* **(I25.8)** with 443 connections or neighbors and so on, as it can be appreciated in the center of the Figure 2 [see Supplementary Datasheet 1 (Network Interactions) and Supplementary Figure 1 (Network Visualizations) for more detail].

The structure of this comorbidity network is such that these highly connected diseases are indeed forming multimorbidity clusters *centralizing* a large fraction of the network comorbidity connections (relatively high network centrality = 0.462). More clear details on multimorbidity patterns may be discussed later, in the context of age-grouped comorbidity networks.

Perhaps the most useful result for clinicians is presented in the comorbidity networks presented in the form of Supplementary Tables [see Supplementary Datasheet 1 (Network Interactions)]. A clinician may go to the table, search for their condition of interest and look up what are the common (or even uncommon) comorbidities for this condition. Many of them will be quite obvious to someone involved in clinical practice. Some others may not and we envision this information may be useful for differential diagnostics or to anticipate complications that may indeed require specialized treatment, say in intensive care units.

### 3.3. Cardiovascular Comorbidities Modules (CVCmodules)

From the CVC network, a *modular decomposition* was performed. Comorbidity modules approach is used to study interrelationships between groups or classes of diseases. We extracted these CVCmodules using a *community detection algorithm* (based on *random walks and information theory*) focused on the interdependence of the links called *Infomap* (see Methods) that resorts the use of the so-called *MapEquation* (Rosvall and Bergstrom, 2008; Bohlin et al., 2014). This model offers a *module-based description* of the emergence gene of comorbidity relations, many of them involve complex or polygenic disorders (Goh et al., 2007).

**Figure 3:**
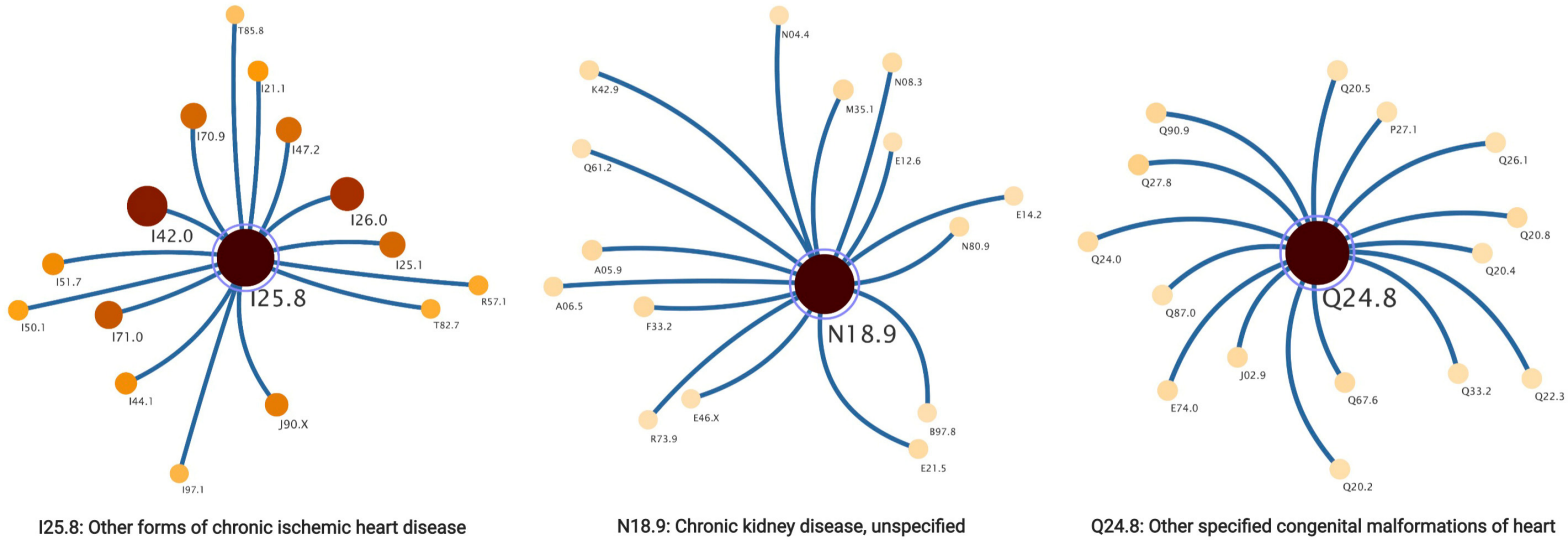
Communities correspond to the most common connected comorbidities.

With illustrative purposes, we show three groups of comorbidities occurring together more often than would have been expected by chance see Figure 3. Each group included between two and eight different comorbidity sub-clusters. We selected those three communities of comorbidities: *Other forms of chronic ischemic heart disease* **(I25.8)**; *Chronic kidney disease, unspecified* **(N18.9)** and *Other specified congenital malformations of heart* **(Q24.8)**, as these modules represent three main areas of interest in cardiovascular health: congenital conditions, purely cardiovascular/circulatory diseases and systemic failures.

Community *Other forms of chronic ischemic heart disease* **(I25.8)** was the largest, it is connected with 711 comorbidities, of which only 307 (43% of comorbidities in this community) were identified with associated genes. In module *Chronic kidney disease, unspecified* **(N18.9)**, 178 related comorbidities were found, but only 66 (37%) of these had associated genes. Regarding the third module selected, *Other specified congenital malformations of heart* **(Q24.8)** was related to 155 diseases and only 67 (43%) had genes associated with them.

### 3.4. Cardiovascular Comorbidity Modules: Physiological, Functional, and Genetic Associations (CVCgenes and CVCpathways)

We prepared a curated mapping of ICD-10 codes based on the genetic disorders reported in ClinVar to describe the relation of metabolic pathways involved in each community diseases selected. Regarding the analysis of the metabolic pathways involved and shared between comorbidities, we analyzed some of the most representative with respect to the Jaccard index (see Methods).

**Figure 4:**
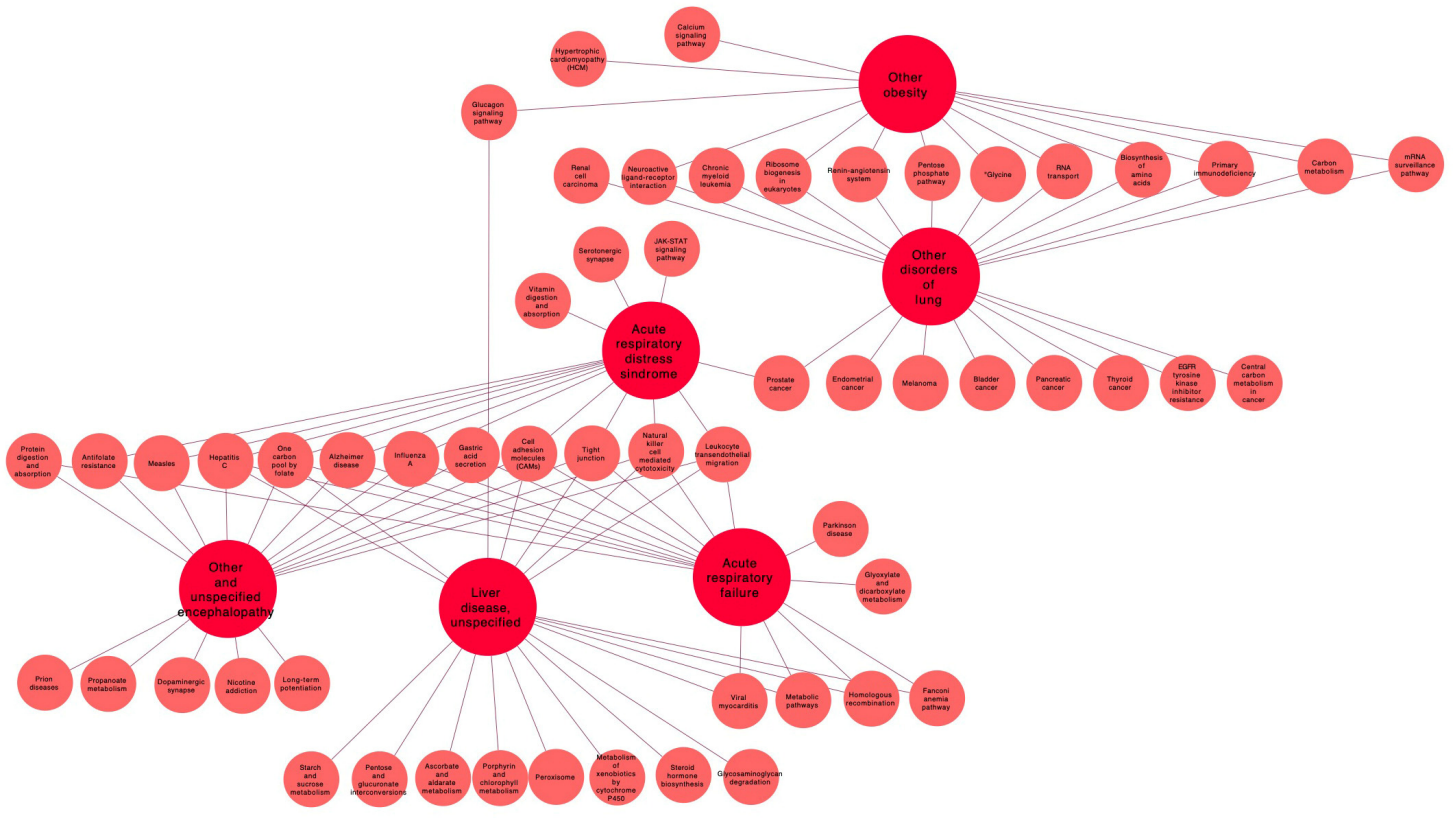
Pathways related to the main I25.8 (other forms of chronic ischemic heart disease) comorbidities.

From *Other forms of chronic ischemic heart disease* **(I25.8)** we choose to analyze in more detail the following comorbidity pairs *Other and unspecified encephalopathy* **(G93.4)** and *Acute respiratory distress syndrome* **(J80.X)**, *Other and unspecified encephalopathy* **(G93.4)** and *Acute respiratory failure* **(J96.0)**, *Other obesity* **(E66.8)** and *Other disorders of the lung* **(J98.4)**, and *Liver disease, unspecified* **(K76.9)** and *Acute respiratory failure* **(J96.0)** (see Figure 4 and Table 1).

**Table 1:** Comorbidities analyzed in the Other forms of chronic ischemic heart disease (I25.8) module.

**Module**	**Comorbidity duplex**	**Common genes**	**Jaccard index**	**Enriched pathways**
Other forms of chronic				
ischemic heart				Folate-mediated one-carbon metabolism
disease (I25.8)				Antifolate resistance
				Cell adhesion
	Other and unspecified			Transendothelial leukocyte migration
	encephalopathy (G93.4)			Tight junction regulation
	and Acute respiratory			Response to hepatitis C
	distress syndrome (J80.X)	437	0.827651515	Natural killer cell-mediated cytotoxicity
				Response to measles
				Alzheimer's disease
				Response to Influenza
	Other and unspecified			Folate-mediated one-carbon metabolism
	encephalopathy (G93.4)			Antifolate resistance
	and Acute respiratory			Cell adhesion
	and failure (J96.0)	440	0.787119857	Transendothelial leukocyte migration
				Tight junction regulation
				Response to hepatitis C
				Natural killer cell-mediated cytotoxicity
				Response to measles
				Alzheimer's disease
				Response to Influenza
				Protein digestion and absorption
	Other obesity (E66.8)			Pentose phosphate pathway
	and Other disorders			Glycine, serine and threonine metabolism
	of lung (J98.4)	1198	0.670772676	Amino acid biosynthesis
				Carbon metabolism
				Primary immunodeficiencies
				Renin-angiotensinogen system
				Neuroactive ligand-receptor interaction
				mRNA surveillance pathway
				RNA transport
				Ribosome biogenesis in eukaryotes
	Liver disease, unspecified			Reserve of one-carbon by folate
	(K76.9) and Acute			Fanconi anemia
	respiratory failure (J96.0)	457	0.640953717	Homologous recombination
				Viral myocarditis
				Cell adhesion molecules (CAM)
				Tight junction
				Transendothelial leukocyte migration
				Cytotoxicity mediated by natural killer cells
				Hepatitis C
				Metabolic pathways

**Figure 5:**
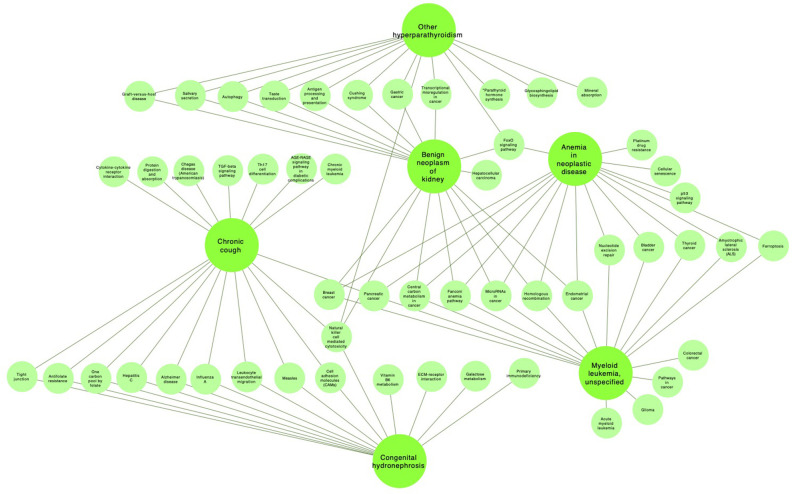
Pathways related to the main N18.9 (chronic kidney disease, unspecified) comorbidities.

Regarding the community of *Chronic kidney disease, unspecified* **(N18.9)**, the pathways of the three comorbidities were compared as it can be seen in Figure 5 and Table 2. *Congenital hydronephrosis* **(Q62.0)** and *Cough* **(R05.X)**, *Benign neoplasm of kidney* **(D30.0)** and *Other hyperparathyroidism* **(E21.1)**, and *Myeloid leukemia, unspecified* **(C92.9)** and *Anemia in neoplastic disease* **(D63.0)**.

**Table 2:** Comorbidities analyzed in the Chronic kidney disease, unspecified (N18.9) module.

**Module**	**Comorbidity duplex**	**Common genes**	**Jaccard index**	**Enriched pathways**
Chronic kidney disease				
unspecified (N18.9)	Congenital hydronephrosis			Cell adhesion molecules
	(Q62.0) and Cough (R05.X)	437	0.640953717	Transendothelial leukocyte migration
				Tight junction
				Natural killer cell mediated cytotoxicity
				Reserve of one-carbon by folate
				Antifolate resistance
	Benign neoplasm of			Processing and antigen presentation
	kidney (D30.0) and Other			Graft-vs.-host disease
	hyperparathyroidism (E21.2)	51	0.467889908	Natural killer cell-mediated cytotoxicity
				Transcriptional dysregulation in cancer
				Gastric cancer
				FoxO signaling pathway
				Autophagy
				Taste transduction
				Salivary secretion
				Cushing's syndrome
	Myeloid leukemia,			Thyroid cancer
	unspecified (C92.9) and			Pancreatic cancer
	Anemia in neoplastic			Bladder cancer
	disease (D63.0)	10	0.185185185	Endometrial cancer
				Breast cancer
				Fanconi
				ALS
				Homologous recombination
				Nucleotide excision repair

**Figure 6:**
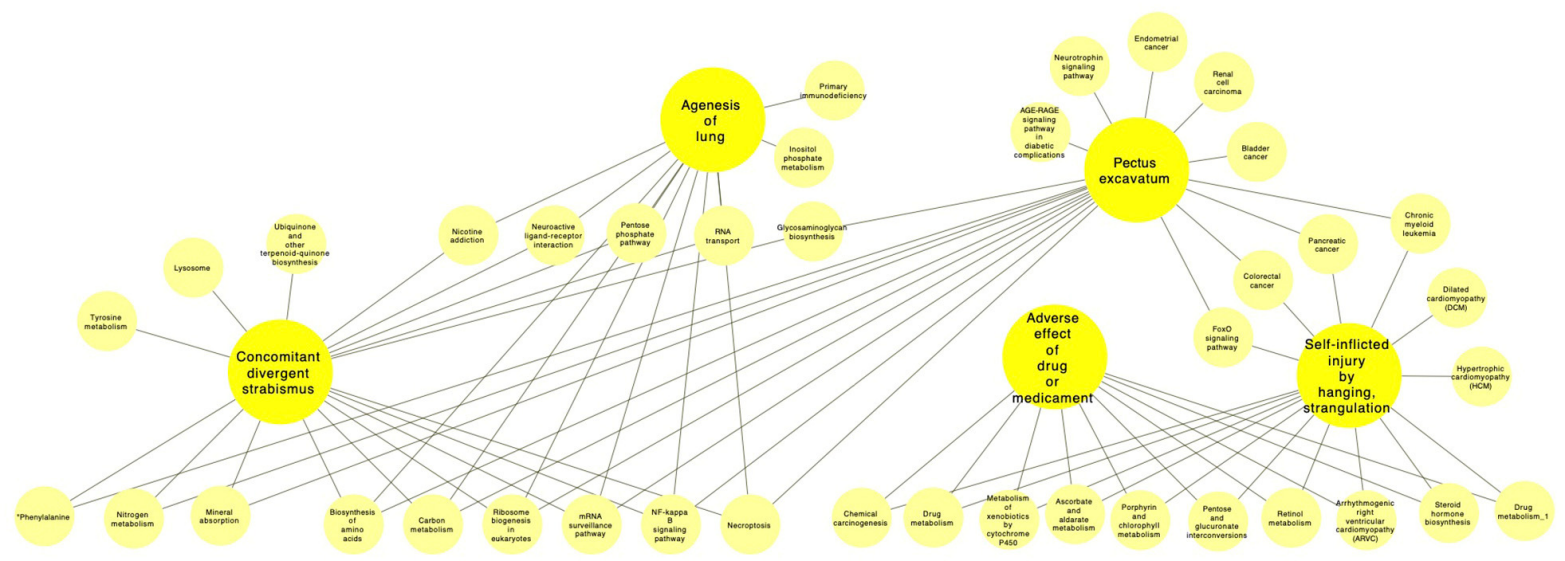
Pathways related to the main Q24.8 (other specified congenital malformations of heart) comorbidities.

For the following *Other specified congenital malformations of heart* **(Q24.8)** community, three comparisons were made: the first between *Exotropia* **(H50.1)** and *Pectus excavatum* **(Q67.6)**, *Exotropia* **(H50.1)** and *Agenesis of lung* **Q33.3** and *Unspecified adverse effect of drug or medication* **(T88.7)** and *Intentional self-inflicted injury by hanging, strangulation or suffocation, at an unspecified location* **(X70.9)**, see Figure 6 and Table 3.

**Table 3:** Comorbidities analyzed in the Other specified congenital malformations of heart (Q24.8) module.

**Module**	**Comorbidity duplex**	**Common genes**	**Jaccard index**	**Enriched pathways**
Other specified				
congenital				Biosynthesis of phenylalanine tyrosine Biosynthesis of tryptophan
malformations				Biosynthesis of glycosaminoglycan
of heart (Q24.8)	Exotropia (H50.1) and			Mineral absorption
	Pectus excavatum (Q67.6)	1,292	0.596766744	Nitrogen metabolism
				Biosynthesis of aminoacids
				Carbon metabolism
				Nf-kappa b signaling pathway necroptosis
				RNAm surveillance
	Exotropia (H50.1) and			Nicotine addiction
	Agenesis of lung (Q33.3)	644	0.37771261	Pentose-phosphate pathway
				mRNA surveillance
				Ribosome biogenesis in eukaryotes
				Amino acid biosynthesis
				Carbon metabolism
				RNA transport
				Nf-kappa b signaling
				Neuroactive ligand-receptor interaction
				Necroptosis
	Unspecified adverse effect			Ascorbate and aldarate metabolism
	of drug or medication (T88.7)			Pentose and glucuronate interconversions
	and Intentional self-inflicted			Steroid hormone biosynthesis
	injury by hanging, strangulation			Retinol metabolism
	or suffocation, in an			Porphyrin and chlorophyll metabolism
	unspecified location (X70.9)	28	0.120689655	Drug metabolism
				Chemical carcinogenesis
				Metabolism of xenobiotics
				by cytochrome P450
				Arrhythmogenic right
				Ventricular cardiomyopathy

On the other hand, a large number of disease pairs that indeed share genetic background, do not show significant comorbidity within our cohort. We may hypothesize that pleiotropy could be playing a role, an observation that has been already made in this regard (Park et al., 2009).

### 3.5. Age-Grouped Cardiovascular Comorbidity Networks (ACVCnetworks)

Since comorbidity and multimorbidity patterns in cardiovascular diseases are highly heterogeneous among different age-groups, it is considered advantageous to analyze comorbidity networks according with said age-groups. In this section, we will present the main results of the analysis of comorbidities as they are present within age-groups spanning over decades, from 0 to 10 years old, 11 to 20, and so on up until 90–100 years of age. The comorbidity network structures and the main players are indeed quite different between age groups. The comorbidity paired tables representing all the networks as well as network visualizations for all of them are included in the Supplementary Figure 1.

**Figure 7:**
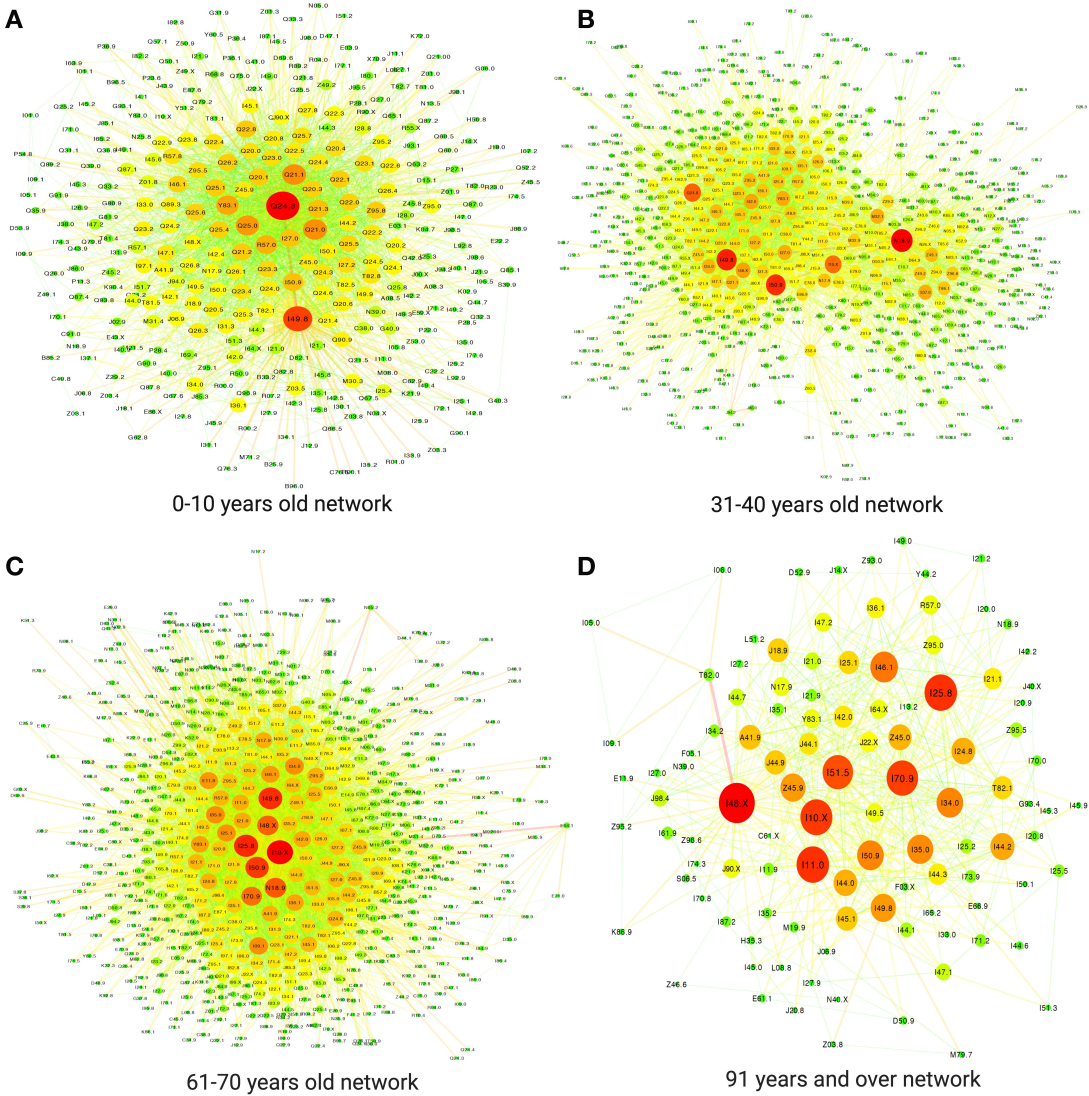
Some examples of groups of ages. **(A)** 0–10 years old network. **(B)** 31–40 years old network. **(C)** 61–70 years old network. **(D)** 91 years and over network.

To exemplify and discuss such differences, in Figure 7 we present a figure with four distinctive age groups: 0–10 (Panel A), 31–40 (Panel B), 61–70 (panel C), and 91–100 (Panel D) years old. As it can be observed in the figure, the topological structure of all four networks is quite different. The network in Panel A corresponding to infancy and early childhood presents a star-like topology in which most connections are dominated by a central node; in this case *Other specified congenital malformations of heart* **(Q24.8)**. Q24.8 is followed, but not-so closely by *Other specified cardiac arrhythmias* **(I49.8)** and most other nodes are in comparison less-connected.

Let us recall that in Figure 7 the sizes and colors of the nodes (diseases) correspond with their connectivity, hence big red nodes are highly connected diseases implying a large number of comorbidities in that age-group, whereas small, green nodes correspond to less connected diseases (i.e., lower number of comorbidities). This star-like topology is also represented in the high value of network centralization = 0.771 for this age bracket as it can be seen in Table 4.

**Table 4:** Comorbidity network topological features by age group.

**Age** **bracket**	**Nodes**	**Links**	**Network** **density**	**Average** **degree**	**Network** **centralization**
0–10	345	2,657	0.045	15.403	0.771
11–20	480	2,955	0.026	12.313	0.467
21–30	625	4,060	0.021	12.992	0.458
31–40	651	4,464	0.021	13.714	0.397
41–50	654	5,098	0.024	15.590	0.383
51–60	681	6,274	0.027	18.426	0.396
61–70	639	6,099	0.030	19.089	0.445
71–80	548	5,032	0.034	18.365	0.405
81–90	326	2,703	0.051	16.583	0.425
91–100	107	506	0.089	9.458	0.351
All ages	1,473	20,543	0.019	27.893	0.462

**Figure 8:**
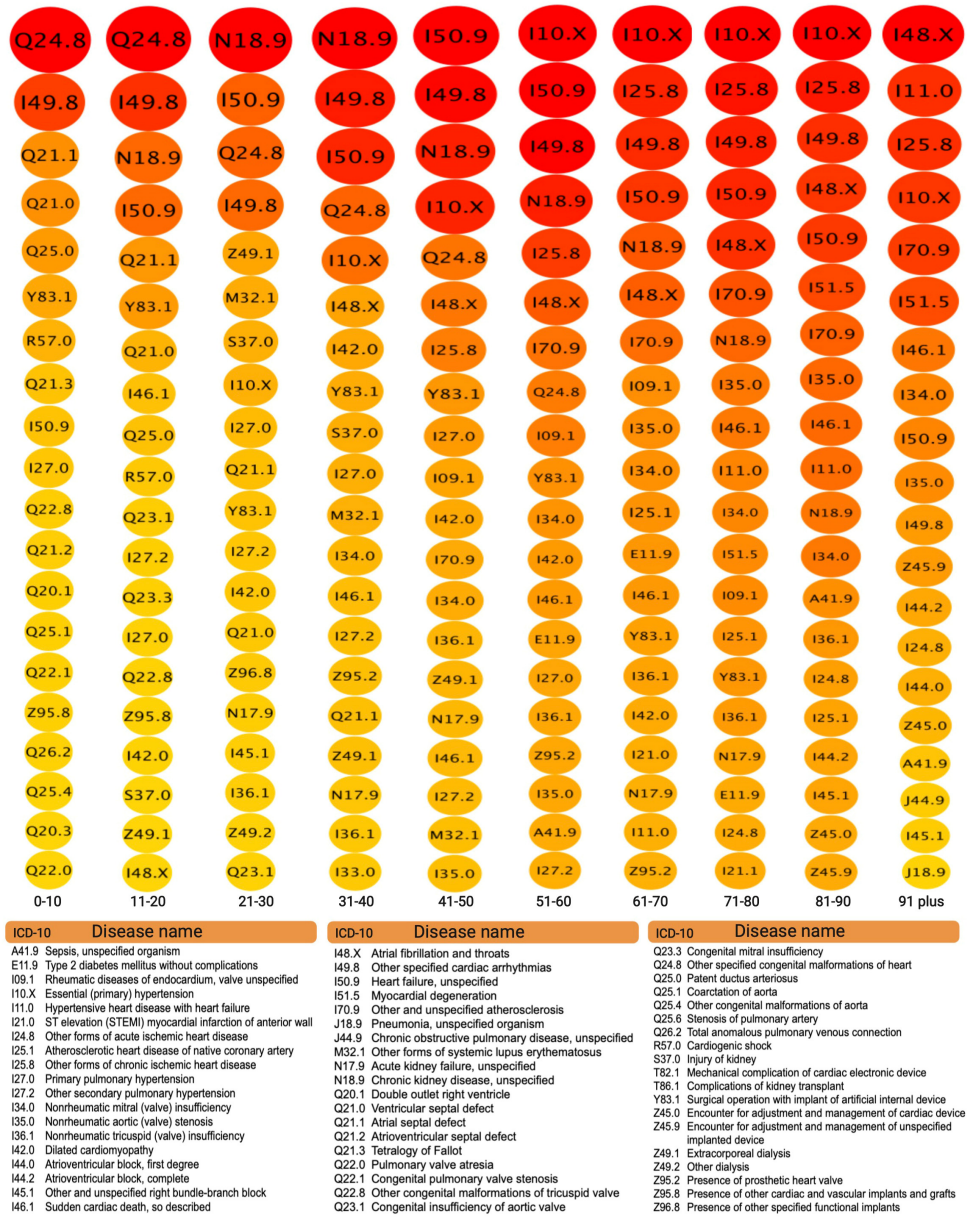
Top 20 connected comorbidities by age group.

In this regard, Figure 8 displays the top20 more connected diseases (the ones with a larger number of comorbidities) for each age group. The figure presents these also by visualizing the degree of connectedness as proportional to size and color, following the same scheme as in Figure 7, i.e., big red nodes are highly connected diseases and small, green nodes correspond to less connected diseases.

Moving on to Figure 7B, corresponding to the comorbidity network for people 31–40 years old. We can notice an important change in network topology with a larger number of highly connected diseases making up for most of the connections in a more *distributed* connectivity, with a network density of 0.021 (similar to the network in panel A) and a network centrality of 0.397 (much lower to the one in the network in panel A) as it can be seen in the corresponding row in Table 4.

Aside from topology, important differences between the comorbidity network for infants and children (Figure 7A) and that of adults aged 31–40 (Figure 7B) lies in the ailments involved. The network for children is mainly centered in birth-related defects, such as congenital malformations and arrhythmias, whereas main players in the adult network in panel B correspond to *sistemic* diseases, such as *Chronic kidney disease, unspecified* **(N18.9)**, *Heart failure, unspecified* **(I50.9)** and *Essential (primary) hypertension* **(I10.X)**, see Figure 8. This systemic character of cardiovascular diseases in the adulthood is indeed captured by the topology of the associated comorbidity network. Connections are more evenly distributed among a larger number of central diseases, a sign of complex traits leading to multimorbidity.

A similar comment can be made regarding (Figure 7C) corresponding to network comorbidities in senior adults aged 61–70 years old. Indeed, it can be argued that network topology points out to a higher presence of multimorbidity (higher network density of 0.03 and middle-valued network centrality of 0.445, as it can be seen in Table 4). After looking up to the corresponding column in Figure 8 we can see that aside from hypertension and chronic kidney diseases **(I10.X and N18.9)**, systemic diseases, such as *Other forms of chronic ischemic heart disease* **(I25.8)** and of the coming back of *Other specified cardiac arrhythmias* **(I49.8)** are connected in a denser complex pattern.

Figure 7D presents again signs of fewer diseases dominating the connectivity distribution, in a much smaller network. Panel D refers to the comorbidity network to individuals more than 90 years old. There are two main cautionary points to be taken into account in the analysis of this particular network: the first is that it corresponds to a significantly smaller set of patients as compared with the other groups [see Supplementary Datasheet 2 (Network Statistics)]. This will of course diminish the heterogeneity captured by the study (although all comorbidities presented are statistically significant). The second is related with the fact that individuals that actually attain such advanced ages (up to 100 years in some cases) are expected to be outliers to a certain degree, meaning that most of them did not suffer greatly from sistemic, chronic degenerative diseases or at least their physical constitution allowed them to survive them.

The aforementioned facts did not preclude the comorbidity network in Figure 7D to be free from complexities. It is the densest of all networks with an impressive 8.9% of all possible comorbidity relations present (see Table 4). This means that although there are fewer diseases present, these tend to accumulate so that any given individual presents a large number of these. This of course may be a truism related with the presence of the organic fragility characteristic of old age.

Regarding the particular composition of the top20 more connected diseases in the 91–100 years old bracket, Figure 8 show them to be mostly related to ischemic and atherosclerotic origins, as expected from accumulated degenerative processes characteristic of the elderly.

## 4. Discussion

We will here discuss the main findings presented in the results regarding the structure of the CVCnetwork, its main structural features as well as what are the hub diseases and core components. In particular, we will show that by analyzing the modular decomposition of the network several highly connected clusters of comorbidities will arise. From the set of CVC modules, we choose three of the larger ones and analyze a number of comorbidity pairs characterized by sharing a common genetic background. In these duplexes, we studied both pathway enrichment patterns for these common genes and clinical evidence of the effects of their comorbidity on human health. Afterwards a brief final considerations subsection has been included to summarize some of the main findings and provide some perspectives of future directions for this kind of research.

### 4.1. Cardiovascular Comorbidity Network (CVCnetwork)

As reported in the Results section, the CVCnetwork is constituted as a single connected component, consistent with previous observations on the complex, interrelated nature of human diseases (Goh et al., 2007). It also comes as no surprise that the most common cardiovascular-related conditions, are the ones more densely connected in the comorbidity network. *Chronic kidney disease, unspecified* **(N18.9)**, *Other specified congenital malformations of heart* **(Q24.8)**, *Other forms of chronic ischemic heart disease* **(I25.8)**, *Essential (primary) hypertension* **(I10.X)** and *Heart failure, unspecified* **(I50.9)** are all complex, system level diseases affecting a lot of physiological and biomolecular processes thus being involved in crosstalk with many diseases. Hundreds of them indeed as it can be seen in Figure 2 and Supplementary Datasheet 1 (Network Interactions).

Indeed, as evidenced by the high average clustering coefficient of the CVCnetwork, and in particular of these “hubs,” such diseases are (as is well-known) also mutually interrelated. However, as we may show in the next subsection there is an intricate but clear modular structure in the CVCnetwork, one in which some of these diseases actually cluster of different *comorbidity modules*, i.e., groups of diseases that not only co-occur more often than by chance but indeed co-occur more strongly than with other comorbidities within the CVCnetwork.

### 4.2. Cardiovascular Comorbidity Modules (CVCmodules)

As it can be seen in the results section, in particular in Figure 3, the CVCnetwork modular partition yields three larger comorbidity modules, formed by between two and eight different comorbidity sub-clusters. These sub-clusters often present a hierarchical submodular structure. For demonstration purposes, we have selected three main comorbidity modules corresponding to *Other forms of chronic ischemic heart disease* **(I25.8)**; *Chronic kidney disease, unspecified* **(N18.9)** and *Other specified congenital malformations of heart* **(Q24.8)**. These modules were chosen by the following criteria: (i) these are highly common diseases in our cohort, (ii) highly interconnected in the CVCnetwork, (iii) distributed in different submodules, (iv) these modules are the larger and richer in structure.

Regarding of the *Other forms of chronic ischemic heart disease* **(I25.8)** module, it contains 711 comorbidities (307 of which have associated genes in the ClinVar database, a fact that will become useful in the next subsection), the *Chronic kidney disease, unspecified* **(N18.9)** module is composed of 178 comorbidities (66 of them with associated genetic origins in ClinVar) and the *Other specified congenital malformations of heart* **(Q24.8)** module which is formed by 155 diseases (67 of which have genes annotated in ClinVar). As presented in Methods, we will further analyze how such clustered comorbidity relations are (partially) related to their genetic origins as well as to its physiological features. To account for genetic background relatedness we will study pairs of diseases clustered in the same module, from their ClinVar annotations we calculated the *Jaccard index* (JI) that points out to the fraction of associated genes they share (see Methods).

#### 4.2.1. Main Comorbidities in the Other Forms of Chronic Ischemic Heart Disease (I25.8) Module

For this module we choose the four comorbidity pairs with the highest Jaccard coefficient for further analysis. The first one of such pairs is formed by *Other and unspecified encephalopathy* **(G93.4)** and *Acute respiratory distress syndrome* **(J80.X)** with 437 common genes (JI = 0.827651515). These apparently disparate diseases share physiological and clinical associations. Burad et al. (2012) found that acute respiratory syndrome in pneumonia patients leads to strong systemic ischemia that may in turn develop into acute encephalopathy. This finding has been further confirmed in a very large (5.6 million cases) epidemiological risk factor study of the group of Bell in the US (Rincon et al., 2014).

Aside from environmental and other risk factors, these diseases large number of shared genes are involved in a number of relevant biomolecular pathways, ranging from *essential metabolism* (folate-mediated one-carbon metabolism, antifolate resistance), *signal transduction* (cell adhesion, transendothelial leukocyte migration, a tight junction regulation). Also, including *immune response and inflammation* (response to hepatitis C, natural killer cell-mediated cytotoxicity, response to measles, Alzheimer's disease, and response to Influenza), as it was evidenced by gene enrichment analysis whose statistical significance was assessed via hypergeometric tests with false discovery rate multiple-testing correction (see Methods).

Closely related to this pair is the second one formed by *Other and unspecified encephalopathy* **(G93.4)** and *Acute respiratory failure* **(J96.0)** with 440 common genes representing a JI = 0.787119857, these genes refer to similar pathways involved with the addition of statistical enrichment of the *protein digestion and absorption* pathway. Understanding the role that such molecular processes may have in the onset and progression of both diseases, of the comorbidity and of their potential multimorbidity relations in the context of the CVCnetwork (see Figure 4), may prove useful, particularly in the design of combined therapeutic strategies with special emphasis in the critically ill patients in intensive care units.

In this regard, we may mention the following: it is known that the physiological manifestation of such biomolecular process starts in the microvascular endothelium (MVE). Abnormal functions of this MVE lead to abnormal haemostasis that may be involved in both encephalopathy and acute respiratory distress, a novel therapy to alleviate this failure from its molecular origins consists in the use of tissue factor pathway inhibitor (TFPI). It has been long known (mostly by animal-based studies) that anti-TF monoclonal antibodies and recombinant TFPI may be helpful to treat these and other conditions (Bajaj and Bajaj, 1997). This comes as no surprise since TFPI therapy works by readjusting these disrupted processes to their homeostatic levels.

On the other hand, in the same module, the comorbidity *Other obesity* **(E66.8)** and *Other disorders of the lung* **(J98.4)**, it was found to share 1,198 genes (JI = 0.670772676). These genes present significant statistical enrichment for the following pathways: Pentose phosphate pathway, Glycine, serine and threonine metabolism, amino acid biosynthesis, and carbon metabolism, all of them related to *essential metabolism*, as well as to primary immunodeficiencies, renin-angiotensinogen system, neuroactive ligand-receptor interaction related to *immune signaling and inflammation*, and to *cell cycle processes*, such as the mRNA surveillance pathway, RNA transport, and ribosome biogenesis in eukaryotes. Although less evident, the physiological relationship between obesity (in particular its inflammatory component) and critical disorders of the lung is not unknown (Bassetti et al., 2011; Pabon et al., 2016; Peters et al., 2018; Szylińska et al., 2018). Under certain circumstances, abnormal immune signals associated with metabolic deregulation may lead to inflammatory proliferation and cytokine storms (Ramos Muniz et al., 2018) that may in turn prove critical for certain functions involved in the lung disease (Lee et al., 2016).

The fourth comorbidity couple discussed in this module involves *Liver disease, unspecified* **(K76.9)** and *Acute respiratory failure* **(J96.0)** (457 shared genes, JI = 0.640953717). These genes present the following enriched pathways: reserve of one-carbon by folate, Fanconi anemia, homologous recombination, viral myocarditis, cell adhesion molecules (CAM), tight junction, transendothelial leukocyte migration, cytotoxicity mediated by natural killer cells, Hepatitis C, and metabolic pathways. We can notice again a lot of immune signaling and transduction activity mediating the crosstalk of these pathologies. Liver disease, for instance, is known to be severely exacerbated by mechanical ventilation (Lai et al., 2020). Lai et al. concluded that in-hospital mortality of patients with high end-stage liver disease scores who required mechanical ventilation was higher and it allowed for predictability of the outcome. The same conclusion was reached previously by Qadir et al. (2018) that traced some of the complications to hepatopulmonary syndrome, portopulmonary hypertension, and hepatic hydrothorax, all of them systemic failures mediated by exacerbated signaling (Karcz et al., 2012; Nuzzo et al., 2019).

#### 4.2.2. Main Comorbidities in the Chronic Kidney Disease, Unspecified (N18.9) Module

Moving onto the second module of interest, the one highly connected with *Chronic kidney disease, unspecified* **(N18.9)**, we will also analyze three pairs of diseases (see Figure 5). We will start by looking at the association between *Congenital hydronephrosis* **(Q62.0)** and *Cough* **(R05.X)**. These seemingly dissimilar diseases share, however 437 common genes (JI = 0.640953717), as in previous cases the molecular associations point out to *immune signaling* (cell adhesion molecules, transendothelial leukocyte migration, tight junction, natural killer cell mediated cytotoxicity) and *essential metabolism* (reserve of one-carbon by folate, antifolate resistance). The joint observation of these diseases, however, presents less dominance than previous cases. It has been reported that about 30% of the cases of congenital hydronephrosis involve definite presence of cough episodes (McHale et al., 1996).

The second duplex, *Benign neoplasm of kidney* **(D30.0)** and *Other hyperparathyroidism* **(E21.2)** involves 51 shared genes (JI = 0.467889908). Overrepresented pathways include *immune responses* (processing and antigen presentation, graft-vs.-host disease, natural killer cell-mediated cytotoxicity), *abnormal transcriptional regulation and cell cycle* (transcriptional dysregulation in cancer, gastric cancer, FoxO signaling pathway, autophagy) as well as *hormone-related signal transduction* (taste transduction, salivary secretion, Cushing's syndrome).

It is noticeable that even if our third comorbidity pair considered in this module *Myeloid leukemia, unspecified* **(C92.9)** and *Anemia in neoplastic disease* **(D63.0)** is, perhaps, one of the most obviously related from the physiological standpoint (Rafei and DiNardo, 2019), does not have such a large genetic background. These diseases only share 10 associated genes, with a mild JI = 0.185185185, general *cancer pathways* (thyroid cancer, pancreatic cancer, bladder cancer, endometrial cancer, breast cancer), *erithopoietic and myeloid differentiation processes* (Fanconi, ALS) and *DNA repair* (homologous recombination, nucleotide excision repair). Not surprisingly, however, the presence of anemia becomes a (bad) prognostic marker in cancer (including of course myeloid leukemia), both in terms of the presence of anemia itself (Paitan et al., 2018) and of the level of circulating myeloblasts (Duong et al., 2018).

#### 4.2.3. Main Comorbidities in the Other Specified Congenital Malformations of Heart (Q24.8) Module

Three comorbidity pairs were also chosen in the *Other specified congenital malformations of heart* **(Q24.8)** module (Figure 6). The first one consists of *Exotropia* **(H50.1)** and *Pectus excavatum* **(Q67.6)** sharing 1,292 genes (JI = 0.596766744). The physiological relationship between these two diseases may be traced to generalized hypotonia and share some similarities with the Elsahy-Waters Syndrome, a rare genetic disease that arise from (mostly biallelic) mutations in the cadherin-11 gene (Castori et al., 2018; Harms et al., 2018). On the genetics and molecular side, both diseases share genes enriched for *metabolic* (biosynthesis of phenylalanine tyrosine and tryptophan, biosynthesis of glycosaminoglycan, mineral absorption, nitrogen metabolism, biosynthesis of aminoacids and carbon metabolism), *signaling* (Nf-kappa b signaling pathway, necroptosis, RNAm surveillance). Interestingly, even if both diseases involve structural dysfunction related for instance to changes connective tissue and collagen formation (Guixiang et al., 2007; Tocchioni et al., 2013a,b; Yao et al., 2016), no statistically significant enrichment was found for related categories in this set of shared genes, suggesting independent molecular mechanisms in both cases.

A similar case arises in the consideration of *Exotropia* **(H50.1)** and *Agenesis of lung* **(Q33.3)** with 644 common genes (JI = 0.37771261), which are involved in nicotine addiction, pentose-phosphate pathway, mRNA surveillance, ribosome biogenesis in eukaryotes, amino acid biosynthesis, carbon metabolism, RNA transport, Nf-kappa b signaling, neuroactive ligand-receptor interaction and necroptosis. There is no reported account of this comorbidity in the published literature, this is unsurprising since both are rare genetic conditions (since they are both rare, finding them associated by chance was quite unlikely, so the fact that we had encountered them in our study group although incidentally fulfilled our statistical criteria, similarly to the previous case with Exotropia and Pectus excavatum). What calls for attention is that they share a relatively high number of genes in functional pathways. This open the way to understanding these rare diseases by studying more common conditions involving similar genes and pathways. Hence, comorbidity networks and functional analyses may help us take a new look at rare genetic conditions and orphan diseases.

A different case is the one involving *Unspecified adverse effect of drug or medication* **(T88.7)** and *Intentional self-inflicted injury by hanging, strangulation or suffocation, at an unspecified location* **(X70.9)**, with 28 common genes, and JI = 0.120689655. Those resulted statistically enriched mostly in *general metabolism* (ascorbate and aldarate metabolism, pentose and glucuronate interconversions, steroid hormone biosynthesis, retinol metabolism) and *drug metabolism* (porphyrin and chlorophyll metabolism, drug metabolism, metabolism of xenobiotics by cytochrome P450, chemical carcinogenesis) as well as in arrhythmogenic right ventricular cardiomyopathy (ARVC). It has been documented that adverse drug events may induce depression or other mental health conditions associated with suicidal thoughts or other forms of self-inflicted damage (Jaga and Dharmani, 2007; Andrew and Brenner, 2015). The particular case of adverse drug effects conducting to attempts of self inflicted hanging or suffocation has been documented in broad scenarios ranging from narcotics (Dinis-Oliveira et al., 2010) to antibiotics (Ahmed et al., 2011).

### 4.3. Cardiovascular Comorbidity Networks by Age (ACVCnetworks)

In this section we will consider some hypothetical clinical case studies to highlight some potential applications of ACVCnetworks as a proof of concept. We will discuss how ACVCnetworks may become an auxiliary tool for differential diagnostics and therapeutics.

- Consider the (hypothetical) case of a 6 years old female patient diagnosed with *Discordant ventriculoarterial connection* **(Q20.3)** as well as a secondary *Supraventricular tachycardia* **(I47.1)**. Prior to surgical interventions, the treating physician team may consider the use of β-blockers, calcium channel blockers and other anti-arrhythmic drugs. However, after consulting the ACVCnetwork for this age (Figure 7A and the full table included in the Supplementary Datasheet 1) they may discover that, aside from a number of congenital defects of the heart and gastroesophageal reflux, *unspecified epilepsy* (**G40.9**) is also common comorbidity of both *Discordant ventriculoarterial connection* **(Q20.3)** and *Supraventricular tachycardia* **(I47.1)**, hence there is an increased probability that their patient may also suffer from it. Care must be taken, then since propranolol for instance, may increase significantly serum thioridazine levels, so it should not be combined with that anti-seizure medications (Yudofsky and Hales, 1992). Selective serotonin reuptake inhibitors may, in turn, increase serum levels of β-blockers; carbamazepine however enhances the opposite effect (Schatzberg et al., 2015). When designing the therapeutic approach with this patient, the team should perhaps consider evaluating some neurological features beforehand.
- When treating an (also hypothetical) 37 years old male patient whose main diagnosis is a *Malignant neoplasm of heart* **(C38.0)** in preparation for the surgical and chemotherapeutic design, it may be worth considering that both, *Pleural effusion* **(J90.X)** and *Chronic kidney disease, unspecified* **(N18.9)** are common comorbidities of heart neoplasms in this age bracket (ACVCnetwork ages: 31–40 depicted in Figure 7B) as reported in the Supplementary Datasheet 1. Special care may be taken when planning the anesthetic and perfusion strategy for the surgery (due to the *Pleural effusion* **(J90.X)** risk) and the type and dose of antineoplastic drugs (to lower its effects on the kidney).
- When evaluating a 65 years old patient with *Specified chronic obstructive pulmonary disease (COPD)* **(J44.8)** also suffering from *Sleep apnea* **(G47.3)** it is of relevance to perform periodic serum and urine sodium tests since *Hyponatremia* **(E87.1)** is a common comorbidity for COPD and for sleep apnea in this age bracket as evidenced by the corresponding ACVCnetwork (Figure 7C and Supplementary Datasheet 1) and it is known that low sodium levels are strongly associated with poor outcomes for COPD patients (Chalela et al., 2016). Respiratory insufficiency in combination with hyponatremia can cause Shy-Drager syndrome that may develop into multiple-system atrophy, a critical, life-endangering condition (Glass et al., 2006).
- An important source of mortality in the elderly (in particular in individuals older than 90 years old) is accidental falls. In some cases traumatisms in the head may lead to *Traumatic subdural hemorrhage* **(S06.5)** (Uno et al., 2017), the management of this disease may be complex in spite of not having such a large number of common comorbidities. In the ACVCnetwork for 91–100 years old (Figure 7D and Supplementary Datasheet 1), we can see that traumatic subdural hemorrhage has only four statistically significant comorbidities in our database. However, by looking at them it is obvious that these may be reason for concern either alone or in the form of multimorbidity. These comorbidities are the following *Atrial fibrillation and flutter* **(I48.X)**, *Hypertensive heart disease with heart failure* **(I11.0)**, *Primary pulmonary hypertension* **(I27.0)**, and *Sepsis* **(A41.9)**. Anyone of such diseases may greatly enhance the risk of dying in elderly subdural hemorrhage patients, an important reason to be aware of comorbidity risks (Hsieh et al., 2018).

### 4.4. Main Findings, Strengths, and Limitations

Careful, systematic examination of electronic health records by means of network and data science approaches is an emerging discipline at the interface of computational biology, biomedical informatics and theoretical medicine. As a nascent research area, it still faces a number of challenges, in turn, it offers a fresh perspective on known problems. Cardiovascular diseases commonly developed into systemic ailments affecting a multitude of organs in different ways, often conducing to multimorbidity and multimortality. For these reasons, the development of an efficient, evidence-based methodology to study comorbidity in cardiovascular diseases is appealing. In this work, we have presented a somewhat straightforward approach to this. The method itself as a tool is a worthy goal. By applying this tool, we were able to discover novel or poorly known features, such as the following.

Comorbidity networks in cardiovascular diseases are highly centralized in the high prevalence diseases, such as cardiac arrhythmias, heart failure, chronic kidney disease, hypertension and ischemic diseases. In spite of this centralized structure, cardiovascular comorbidity networks are actually quite modular on their connectivity. Interestingly (and perhaps expectedly to some extent) modules often recapitulate physiopathological commonalities, for instance, by clustering ischemic diseases with similar ailments. Such is also the case of chronic systemic diseases (kidney, liver, rheumatological diseases, and others), of congenital malformations and others. We have been able to track down the genetic and environmental commonalities behind some of the relations in these modules by resorting to clinical genetics databases and functional pathway enrichment studies.

By looking at some of these modules, we were able to notice how acute respiratory failure related to ischemic heart diseases may complicate with encephalopathy due to their shared molecular and physiological features. Also, how chronic kidney disease share a common genetic background (and a functional one at the level of immune deregulation) with common cough and hydronephrosis. We could also probe on some examples relevant to therapeutic designs, such as evaluating the possible presence of early signs for epilepsy prior to administering β-blockers or calcium channel inhibitors to childhood patients with supraventricular tachycardia; checking for signs of pleural effusion or chronic kidney disease while designing surgical and chemotherapeutic procedures for adult patients with malignant heart neoplasms.

However, aside from these few specific examples, an important contribution of this work lies in the databases created in the form of networked objects. Networks were built for significant comorbidity relationships in cardiovascular and related diseases, both in general and age-specific. These networks are indeed relational databases that may be consulted by clinicians, understood as aids in differential diagnostics or, more commonly to prepare for complications.

Of course, many of these comorbidities are well-known to the practising clinician, perhaps even expected. Other however, may pass unnoticed even to an experienced medical team. It is in those cases that the vast wealth of systematized information, gathered in over almost a decade of treatment on a third-level plus specialized research hospital gains relevance as a data-intensive tool for the clinical practice. Let us recall that the NIC-ICh is a reference institution in cardiovascular diseases, one of Mexico's National Institutes of Health. These databases (networks are included as Supplementary Datasheet 1 in the form of searchable tables) are aimed at constituting a main contribution of this work. On the one hand, this information comes directly from large-scale empirical data, on the other it has been organized in the form of curated, easily-searchable databases.

Large scale, semi-automated databases are not free from limitations: reporting errors, missing data and so on. In the present case an additional limitation lies in the fact that it is based on mining electronic health records from hospital administrative databases. Such databases are built by using the international disease codes. ICD-10 codes are systematic and easy to code and record, however, they do not always capture completely or even accurately the actual complexities of disease and comorbidity.

In the work presented here, cases were from patients discharged from a single health institution specialized in cardiology. This may limit the generalization of our results to other medical specialties. Information from retrospective data on diagnoses of hospital discharges may be also subject to residual bias: The network and community-based analysis of comorbidities conducted in this study is limited to the hospital setting.

The generalizability of the method is indeed relatively straightforward, both for other types of diseases and for regional, national and even international settings provided that:

1. Coding is performed and registered following similar stringent and validated criteria.
2. The number of instances (patient records and comorbidities) is large enough for statistical analysis (hypergeometric tests) to hold.

These are conditions that a medium-to-large, second to third level (and higher) hospital may fulfill but perhaps a small clinic may not. However, the generalization of some of the actual comorbidity relations in different hospital settings may not fully hold since discovery has been made in a quite large but somewhat specialized hospital. By applying similar methodology in local, regional, national and international settings, we envision that even specific comorbidity relationships may be generalizable.

Despite relying on good coding practice for administrative databases, we are aware that it may be subject error sources, as has been reported in other studies using ICD-10. As a cautionary note we have included a full appendix describing the scope and limitations of ICD-10 coding in the Supplementary Document 1. Hence, as valuable a tool as the present work may be it needs to be properly assessed and taken with a lot of caution.

### 4.5. Final Considerations

Cardiovascular diseases are the leading causes of death worldwide, and have been for decades. One source of mortality is the fact that cardiac diseases are often systemic with complex multimorbidity patterns. Analyzing these patterns may advance our own understanding of these illnesses not as a series of isolated events, but as different manifestations of many concurrent causes, both genetic and environmental. Here we have analyzed comorbidity patterns as they have been occurring and reported over 6 years of the full set of admissions to the country-level reference institution for cardiovascular diseases in Mexico, the National Institute of Cardiology “Ignacio Chávez”.

The detailed statistical analysis of almost 35,000 electronic health records allowed us to infer a cardiovascular comorbidity network. We performed modularity analysis of such network to unveil an intricate interconnectedness structure. Choosing a small number of these modules, to exemplify. We analyzed the common genetic background of comorbidity disease pairs as well as their clinical associations and probable risk factors. By continued and consistent analysis of these types of patterns, we envisaged that it may be possible to acquire, strong clinical and basic insights that may further our advance toward a better understanding of cardiovascular diseases as a whole. Hopefully these may in turn lead to further development of better, integrated therapeutic strategies.

## Data Availability Statement

All datasets generated for this study are included in the article/Supplementary Material.

## Author Contributions

EH-L conceived the project. EH-L and MV directed and supervised the project. MM-G and EH-L designed and developed the computational strategy and wrote the manuscript. MM-G and HC-Á implemented the code and database search procedures. MM-G, HC-Á, and EH-L conducted the calculations and validation. MM-G, HC-Á, MV, and EH-L analyzed the results. All authors reviewed and approved the manuscript.

## Conflict of Interest

The authors declare that the research was conducted in the absence of any commercial or financial relationships that could be construed as a potential conflict of interest.
