# Supplementary figures and images for: Comorbidity Networks in Cardiovascular Diseases
Source: Front Physiol. 2020 Aug 28;11:1009. doi: 10.3389/fphys.2020.01009 (PMC7485389; doi:10.3389/fphys.2020.01009)

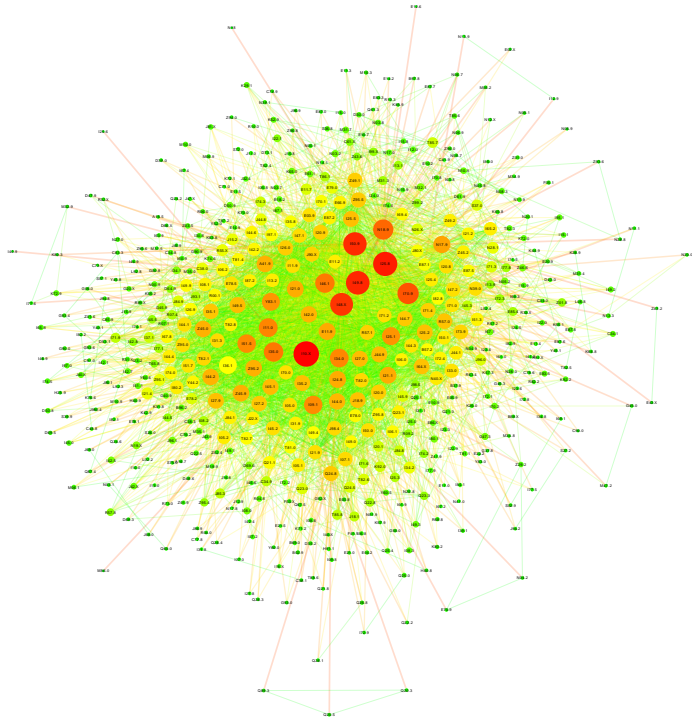

Supplement: Supplementary file 3 [file Data_Sheet_3.ZIP › Supp_Figures_1_Network_Visualizations/FIGnet_71_80.pdf]

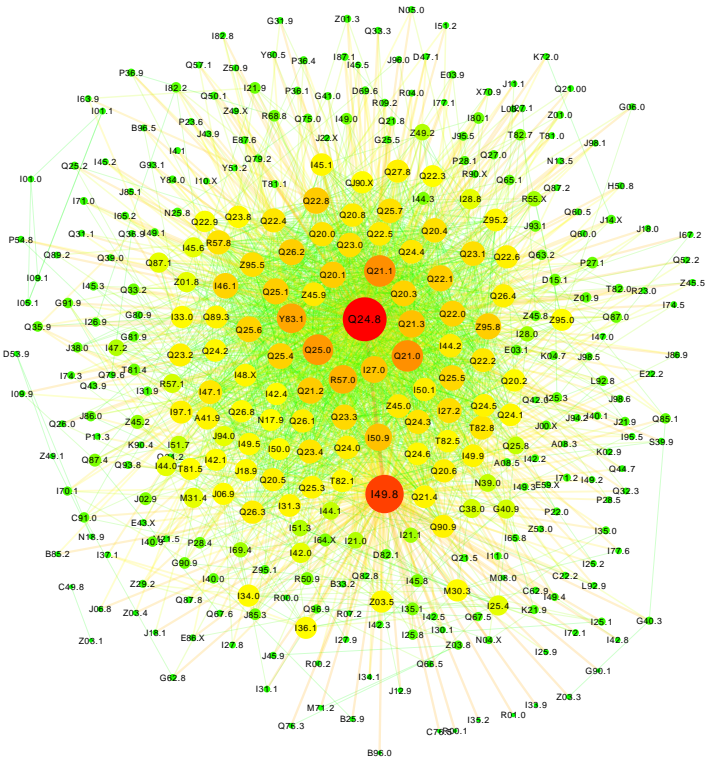

Supplement: Supplementary file 3 [file Data_Sheet_3.ZIP › Supp_Figures_1_Network_Visualizations/FIGnet_0_10.pdf]

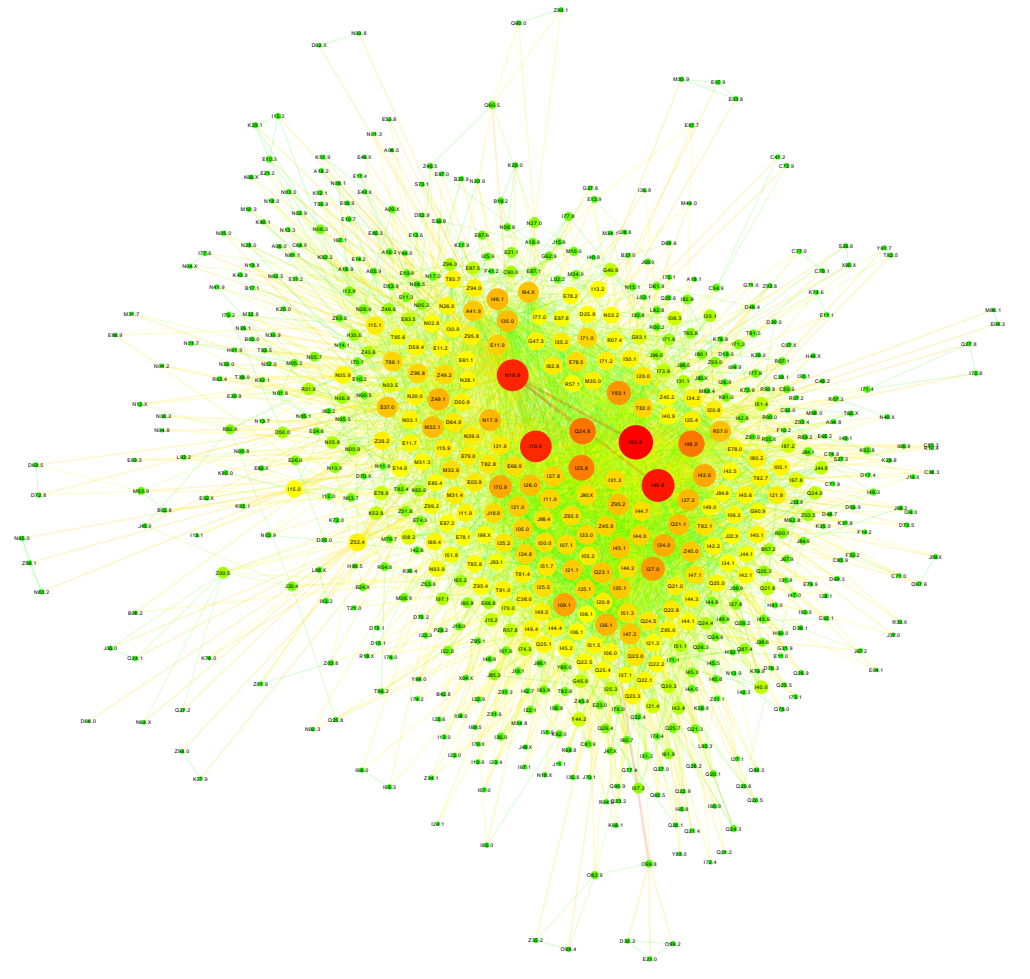

Supplement: Supplementary file 3 [file Data_Sheet_3.ZIP › Supp_Figures_1_Network_Visualizations/FIGnet_41_50.pdf]

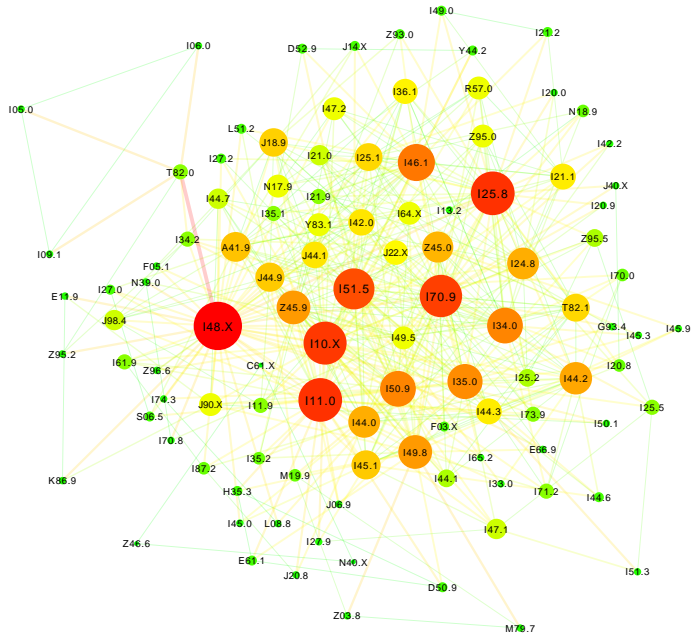

Supplement: Supplementary file 3 [file Data_Sheet_3.ZIP › Supp_Figures_1_Network_Visualizations/FIGnet_90_100.pdf]

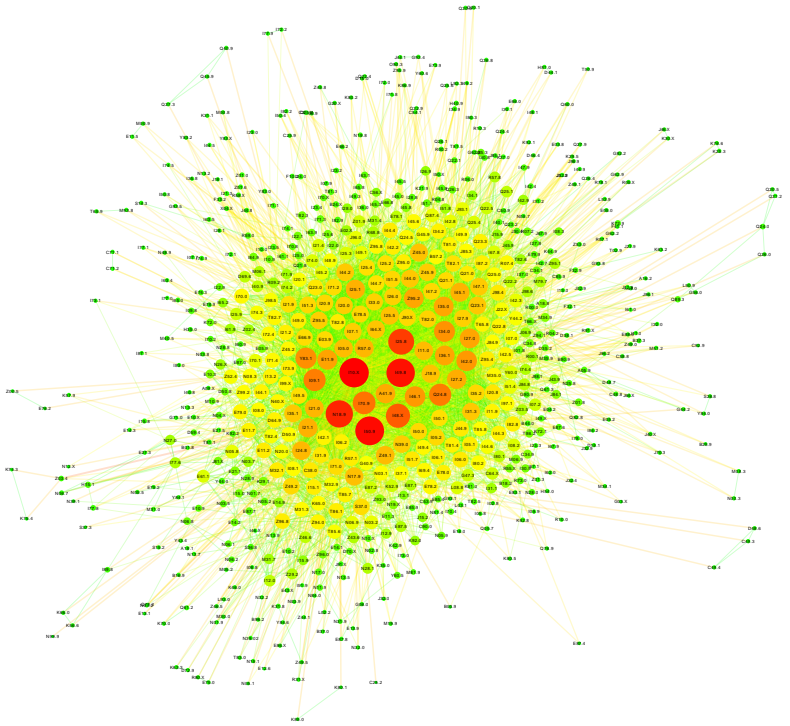

Supplement: Supplementary file 3 [file Data_Sheet_3.ZIP › Supp_Figures_1_Network_Visualizations/FIGnet_51_60.pdf]

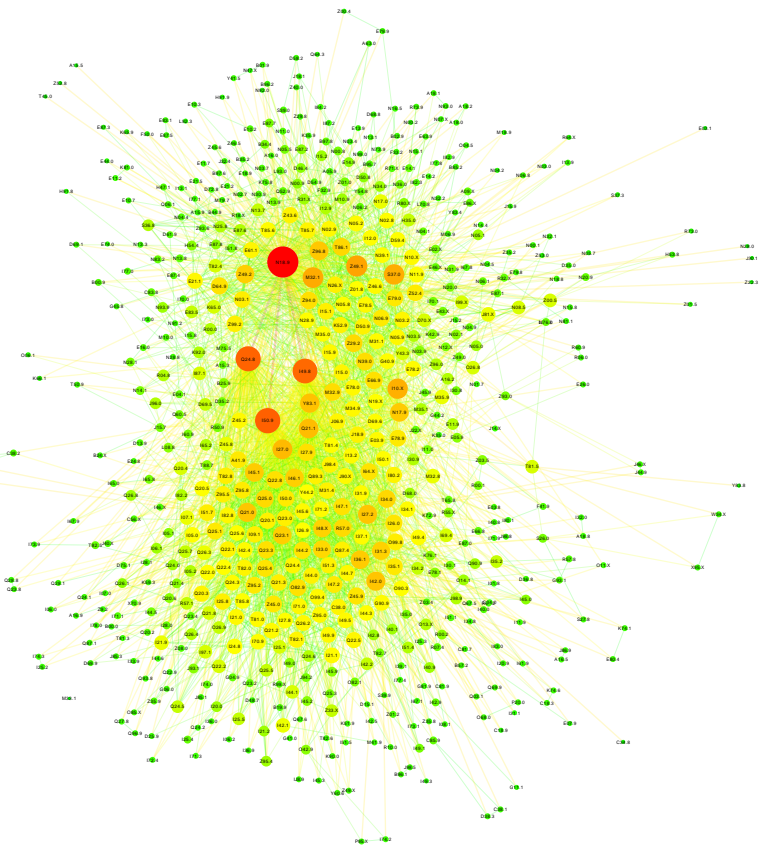

Supplement: Supplementary file 3 [file Data_Sheet_3.ZIP › Supp_Figures_1_Network_Visualizations/FIGnet_21_30.pdf]

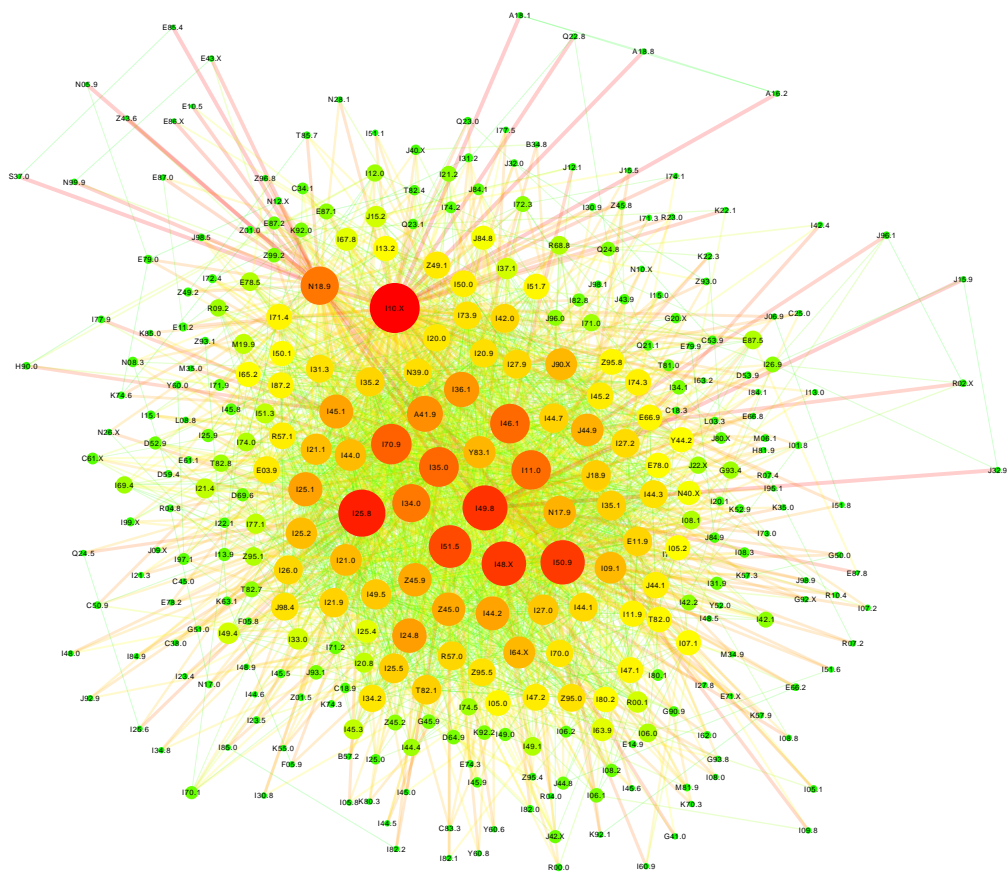

Supplement: Supplementary file 3 [file Data_Sheet_3.ZIP › Supp_Figures_1_Network_Visualizations/FIGnet_81_90.pdf]

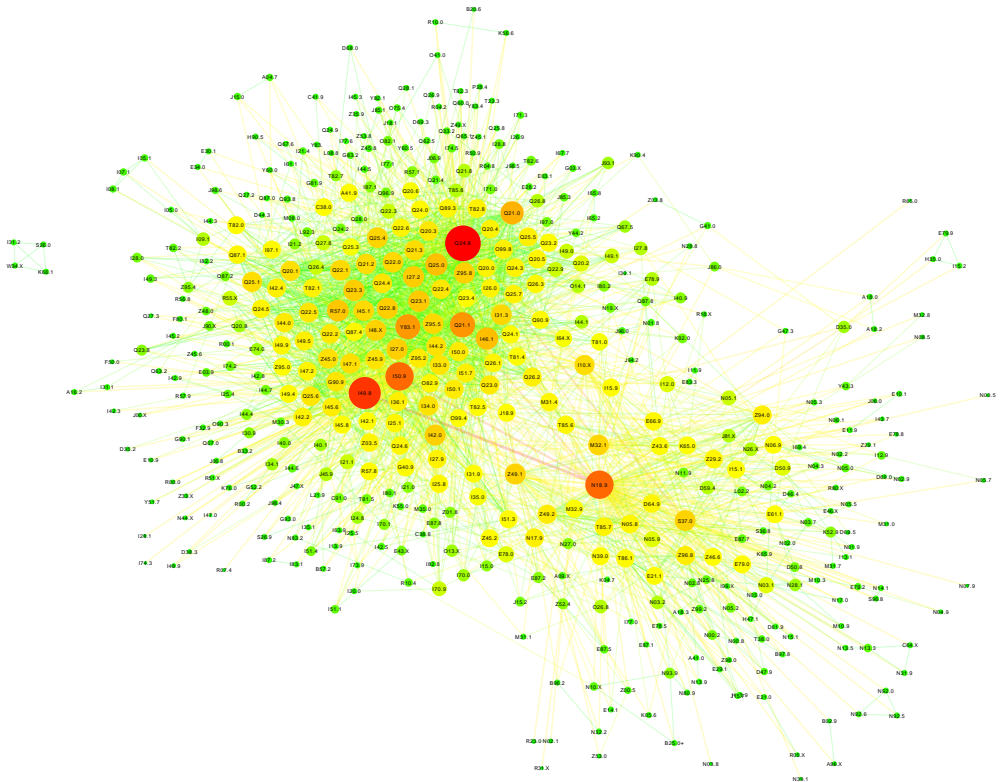

Supplement: Supplementary file 3 [file Data_Sheet_3.ZIP › Supp_Figures_1_Network_Visualizations/FIGnet_11_20.pdf]

N17.2

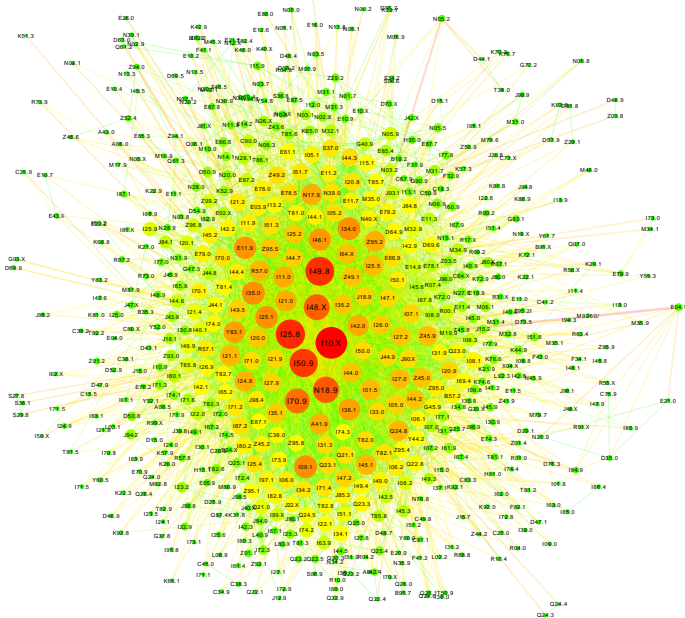

Supplement: Supplementary file 3 [file Data_Sheet_3.ZIP › Supp_Figures_1_Network_Visualizations/FIGnet_61_70.pdf]

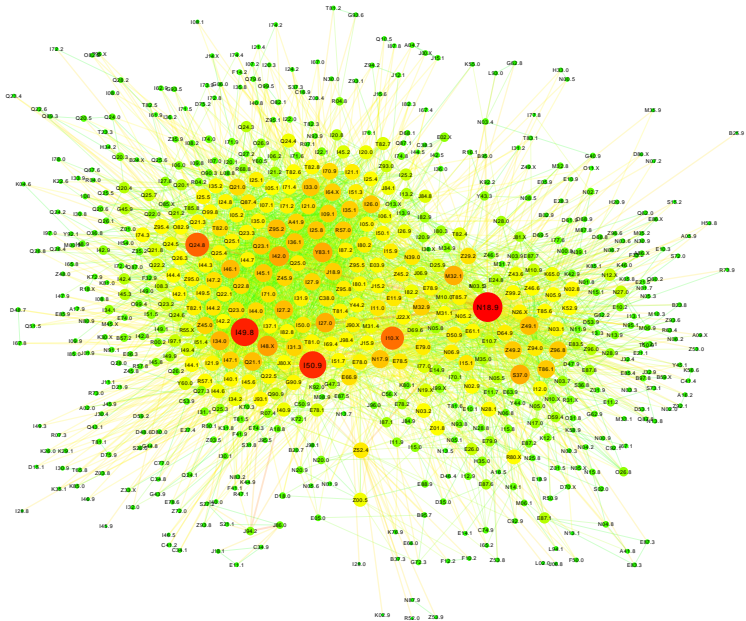

Supplement: Supplementary file 3 [file Data_Sheet_3.ZIP › Supp_Figures_1_Network_Visualizations/FIGnet_31_40.pdf]
