# Supplementary material for: Comorbidity Networks in Cardiovascular Diseases
Source: Front Physiol. 2020 Aug 28;11:1009. doi: 10.3389/fphys.2020.01009 (PMC7485389; doi:10.3389/fphys.2020.01009)
Supplement: Supplementary file 4 [file Data_Sheet_4.PDF]

Comorbidity networks in cardiovascular diseases (Supplementary Appendix 1)

Héctor A. Cruz-Ávila [1,2], Maite Vallejo [2], Mireya Martínez-García[2], and Enrique Hernández-Lemus [3,4,*]

[1] *Autonomous University of México City, México*
[2] *Sociomedical Research Unit, National Institute of Cardiology 'Ignacio Chávez', México*
[3] *Computational Genomics Division, National Institute of Genomic Medicine (INMEGEN), México*
[4] *Centro de Ciencias de la Complejidad, Universidad Nacional Autónoma de México, 04510 Mexico City, Mexico*

Correspondence*:
Enrique Hernández-Lemus
ehernandez@inmegen.gob.mx

ICD-10 CODING

The clinical data is essential for the generation of data necessary for decision-making in healthcare, surveillance and research. This information must be plausible from a statistical analysis (Delgado et al., 2005). A coding process had been necessary in which medical condition be measured has defined and standardized using a controlled vocabulary, such as the World Health Organization's International Classification of Diseases, tenth revision (ICD-10), an administrative hospital discharge data. ICD-10 was introduced worldwide beginning in the late 1990s and has become the international standard diagnostic classification for reporting morbidity and mortality (Jetté et al., 2010).

Having a universally accepted ICD classification has been facilitated comparability of morbidity data internationally, has helped to decrease omissions and errors in administrative data collection (Jetté et al., 2010). When consideration is given to the nature of the analysis, ICD can provide highly reliable population-based estimates of hospitalization rates, for healthcare use but also for epidemiological researched (Henderson et al., 2006).

Also, *clinical modifications to the ICD-10 for hospital use (ICD-10MC)* that contain a specific volume of medical procedures is most recent. It is mandatory to use ICD-10-CM for billing data reporting in the US since 2015 and it is widely used in many European countries. The WHO itself has supported the development of these standard terminology for hospital diagnosis and is maintained by other for example, the Centers for Medicare and Medicaid Services (CMS), Centers for Disease Control and Prevention (CDC), American Hospital Association (AHA) and American Health Information Management Association (AHIMA) (Hernandez-Ibarburu et al., 2019).

However, since the data are primarily collected for administrative or billing purposes, several groups have reported ICD coding errors in inpatient data, so there are underlying concerns about whether they are suitable for other secondary purposes (Henderson et al., 2006; Amy et al., 2017; Burles et al., 2017; Peng et al., 2020).

These concerns are related to several factors to consider (Delgado et al., 2005):

- Health personnel record the diagnosis: When physicians or nurses record the diagnosis, they often do so in unstructured narrative text, and without specifying whether it is an acute or chronic event, or does not indicate the anatomical site (As occurred with the term *sinusitis* in Delgado et al. (Delgado et al., 2005). The condition was assigned interchangeably by the following codes: J01.9 *acute sinusitis, unspecified* (7 times), J02.9 *acute pharyngitis, unspecified* (1 time) and J32.9 *chronic sinusitis, unspecified* (3 occurrences).
- Coding by nosologists: Specialized nosologists staff, generally from the statistics and epidemiology office, encodes medical notes to lessen the ambiguity of the free text. However, this task is usually performed manually, which entails a degree of predictable error, where the code chosen by the digitizer may not represent the clinical situation reported for a patient.
- The health personnel himself encode the event: With the apparent intention of reducing the administrative coding error, the medical providers code the sanitary event, but they generally unaware of all the possible codings existing in CID-10 or don't have time to do that with the precision required for management and health research.
- In any of the above cases, there are two possible sources of error, the *completeness defect* when the event actually occurred is not fully recorded on medical forms and the *accuracy defect* when assigned to an erroneous nomenclature of ICD-10 code. For example, in a service of General Pediatrics and Pediatric Specialties of a public hospital in Peru, a decrease in the quality (validity) of the reported medical information of up to 50.8 % was reported, being one of the most regular problems the use of codes. nonspecific (Delgado et al., 2005).
- Additionally, the ICD-10 does not have a defined classification axis (there are chapters whose axis are etiological, and in others it is anatomical), and it also has some specific gaps (Cohen et al., 2019). For example, the use of the diagnostic term *persistent mild asthma* could not be coded because the ICD-10 in the section corresponding to asthma did not include the degree of severity (Delgado et al., 2005).

The validity of ICD codes for identifying specific conditions depends on whether the condition contributes to health service use, and on where, when, and how data are collected (Peng et al., 2017). Some validation studies have performed a diagnostic accuracy of ICD-10 evaluated according to sensitivity, specificity, positive predictive values (PPV) and negative predictive values (NPV). They have found that ICD-10 codes for specific major diagnoses, major procedures, minor procedures, ambulatory diagnoses, co-existing conditions and death status showed good-to-excellent coding quality.

For example, Henderson et al. audit data from 2 random samples of more than 14,000 hospital discharges, they reported accuracy of ICD-10 for principal diagnosis and principal procedure of 87% and 81% respectively, and concluded that diagnosis and procedure coding maintained a high standard of quality (Henderson et al., 2006). Amy et al. used two models for evaluate ICD-10 to Transient Ischemic Attack among 417 patients. They showed the most restrictive algorithm had the lowest sensitivity (36.8%), but highest specificity (92.5%) and PPV (76.0%). The most inclusive algorithm had the highest sensitivity

(63.8%), but lowest specificity (81.5%) and PPV (68.9%) (Amy et al., 2017). Cohen et al. evaluate ICD-10 for Adult Congenital Heart Disease (ACHD). They found of the 6000 cases, ICD-10 codes correctly categorized 629 as having ACHD, sensitivity 0.81 (95% confidence interval 0.78—0.83), specificity of 0.99 (95% confidence interval 0.99—1) (Cohen et al., 2019).

Luhn et al. explored the feasibility and accuracy of obtaining information on tumor sidedness from electronic health records billing codes of 200 with metastatic colorectal cancer (MCRC) patients (Ruwald et al., 2012). Concordance was determined via observed agreement and Cohen's kappa coefficient (K). The observed agreement between the ICD codes and abstracted data from a tumor site for all sampled patients was 0.58 (K=0.41). When restricting to the 62% of patients with a side-specific ICD code, the observed agreement was 0.84 (K=0.79). The specificity (92–98%) of structured data for tumor location was high, with lower sensitivity (49–63%), PPV (64–92%) and NPV (72–97%). They demonstrate that ICD codes adequately characterize a side of colon for use in studying outcomes for left- versus right-sided colon tumors following specific therapies (Ruwald et al., 2012).

Lawrence et al. (Lawrence et al., 2019) evaluate the accuracy of ICD-10 codes for identifying thromboembolic events occurring during anticoagulation therapy.There were 661 hospitalizations identified among 487 anticoagulated patients. Overall thromboembolic ICD 10 coding sensitivity was 100.0% (95% CI 87.2–100.0); specificity was 79.3% (75.9–82.4). The PPV was 17.1% (11.6–23.9%), and NPV was 100% (99.3–100.0). The authors concluded, ICD-10 codes can reliably be used for ruling out hospitalizations for thromboembolic events in patients receiving anticoagulation therapy, but should not be used for identifying thromboembolic complications without confirmatory chart review (Lawrence et al., 2019).

Burles et al. explored 1453 of patients ambulatory records with a pulmonary embolism (PE) ICD 10 code (Burles et al., 2017). PE diagnoses were confirmed by reviewing medical records and imaging reports. The sensitivity of PE ICD 10 codes in this dataset was 91.1% (95%CI, 89.4–92.6) with a specificity of 99.9% (95%CI, 99.9–99.9). The positive and negative predictive values were 82.3% (95%CI, 80.3–84.2) and 99.9% (95%CI, 99.9–99.9), respectively. Also, they concluded that ambulatory care data, like inpatient data, are subject to coding errors. This confirms the importance of ICD- 10 code validation prior to use (Burles et al., 2017).

Chi et al. assessed the validity of ICD-10-CM codes to discriminate between type 1 diabetes mellitus (T1DM) and type 2 diabetes mellitus (T2DM) among health plan members with youth-onset (diagnosis, age <20 years) diabetes (Chi et al., 2019). The gold standard for diabetes type is the physician-assigned diabetes type documented in patients' medical records. According to the gold standard, 1911 persons had T1DM and 652 persons had T2DM (mean age (SD): 19.1 (6.5) years). They obtained 90.6% (95% CI 88.4% to 92.9%) sensitivity, 96.3% (95% CI 95.4% to 97.1%) specificity, 89.3% (95% CI 86.9% to 91.6%) PPV, 96.8% (95% CI 96.0% to 97.6%) NPV, and 94.8% (95% CI 94.0% to 95.7%) accuracy for discriminating T2DM from T1DM. The authors concluded that ICD-10-CM codes can accurately classify diabetes type for persons with youth-onset diabetes, showing promise for rapid, cost-efficient diabetes surveillance (Chi et al., 2019).

Regardless coding of certain secondary diagnosis, Rattanaumpawan et al. determined the accuracy of the ICD-10 coding system in identifying comorbidities and infectious conditions using data from a Thai university hospital administrative database (Pinyo Rattanaumpawan et al., 2016). Patient comorbidities were captured using the ICD-10 coding algorithm for the Charlson comorbidity index. Conditions with ICD-10 codes that had good sensitivity (90% or higher) were diabetes mellitus and HIV infection. They reported the conditions with ICD 10 codes that had good specificity (90% or higher) were cerebrovascular disease, chronic lung disease, diabetes mellitus, cancer, HIV infection, and all infectious conditions. By combining ICD 10 codes with microbiological results, sensitivity increased from 49.5 to 66% for UTI and from 78.3 to 92.8% for BSI (Pinyo Rattanaumpawan et al., 2016).

Chart abstractions by researchers are guided by clinical definitions of disease, which may differ from coding standards, whereas comparisons to clinical databases may use information not limited to the chart. Both of these factors may explain the differences in the range of sensitivities found in the studies (Henderson et al., 2006).

Incidence and prevalence estimates based on administrative data may be biased and a validation of the codes used in the analysis may need to be undertaken. This may be particularly important for diseases and procedures which are considered uncommon or minor. In contrast, administrative data appear to produce quite robust results in analytic studies when the misclassification of diagnoses (particularly comorbidities) must be quite extreme to bias the results (Henderson et al., 2006). Although validation studies can be carried out to ensure the validity of coding in administrative data, there are still limitations when using ICD data (Jetté et al., 2010).

THE MEXICAN SCENARIO

The Mexican Center for Classification of Diseases and Collaborating Center for the WHO Family of International Classifications in Mexico (CEMECE), is the national center of reference to promote and monitor the correct use of the International Classifications of the World Health Organization (WHO) in Mexico, including the International Statistical Classification of Diseases and Health-Related Problems, Tenth Revision (ICD-10). CEMECE is responsible for training and updating medical information coders and coder instructors who use the ICD in public and private medical institutions. The coding of hospital morbidity was extended to the entire public health system from 1995. The implementation of the ICD-10 in Mexico in 1998 gave an additional impetus to increase the training of coders and extend its application in the private sector.

REFERENCES

Amy, Y., Quan, H., McRae, A., Wagner, G. O., Hill, M. D., and Coutts, S. B. (2017). Moderate sensitivity and high specificity of emergency department administrative data for transient ischemic attacks. *BMC health services research* 17, 666

Burles, K., Innes, G., Senior, K., Lang, E., and McRae, A. (2017). Limitations of pulmonary embolism icd-10 codes in emergency department administrative data: let the buyer beware. *BMC medical research methodology* 17, 89

Chi, G. C., Li, X., Tartof, S. Y., Slezak, J. M., Koebnick, C., and Lawrence, J. M. (2019). Validity of icd-10-cm codes for determination of diabetes type for persons with youth-onset type 1 and type 2 diabetes. *BMJ Open Diabetes Research and Care* 7

Cohen, S., Jannot, A.-S., Iserin, L., Bonnet, D., Burgun, A., and Escudié, J.-B. (2019). Accuracy of claim data in the identification and classification of adults with congenital heart diseases in electronic medical records. *Archives of cardiovascular diseases* 112, 31–43

Delgado, R. P., Zavalaga, L. F. L., Morales, E. A. C., and Garcia, L. L. (2005). Concordancia entre el diagnóstico médico y la codificación de informática, considerando el cie-10, en la consulta externa de pediatria en el hospital nacional cayetano heredia, lima-perú. *Revista Médica Herediana* 16, 239–245

Henderson, T., Shepheard, J., and Sundararajan, V. (2006). Quality of diagnosis and procedure coding in icd-10 administrative data. *Medical care* , 1011–1019

Hernandez-Ibarburu, G., Perez-Rey, D., Alonso-Oset, E., Alonso-Calvo, R., de Schepper, K., Meloni, L., et al. (2019). Icd-10-cm extension with icd-9 diagnosis codes to support integrated access to clinical legacy data. *International journal of medical informatics* 129, 189–197

Jetté, N., Quan, H., Hemmelgarn, B., Drosler, S., Maass, C., Oec, D.-G., et al. (2010). The development, evolution, and modifications of icd-10: challenges to the international comparability of morbidity data. *Medical care* , 1105–1110

Lawrence, K., Joos, C., Jones, A. E., Johnson, S. A., and Witt, D. M. (2019). Assessing the accuracy of icd-10 codes for identifying acute thromboembolic events among patients receiving anticoagulation therapy. *Journal of thrombosis and thrombolysis* 48, 181–186

Peng, M., Lee, S., D'Souza, A. G., Doktorchik, C. T., and Quan, H. (2020). Development and validation of data quality rules in administrative health data using association rule mining. *BMC Medical Informatics and Decision Making* 20, 1–10

Peng, M., Southern, D. A., Williamson, T., and Quan, H. (2017). Under-coding of secondary conditions in coded hospital health data: impact of co-existing conditions, death status and number of codes in a record. *Health informatics journal* 23, 260–267

Pinyo Rattanaumpawan, M., Wongkamhla, T., and Thamlikitkul, V. (2016). Accuracy of icd-10 coding system for identifying comorbidities and infectious conditions using data from a thai university hospital administrative database. *J Med Assoc Thai* 99, 368–73

Ruwald, M., Hansen, M., Lamberts, M., et al. (2012). Accuracy of the icd-10 discharge diagnosis for syncope [published online ahead of print november 4, 2012]. *Europace*
