# Supplementary material for: Comorbidity Networks in Cardiovascular Diseases
Source: Front Physiol. 2020 Aug 28;11:1009. doi: 10.3389/fphys.2020.01009 (PMC7485389; doi:10.3389/fphys.2020.01009)
Supplement: Supplementary file 5 [file Data_Sheet_5.PDF]

Comorbidity networks in cardiovascular diseases (Supplementary Appendix 2)

Héctor A. Cruz-Ávila [1,2], Maite Vallejo [2], Mireya Martínez-García[2], and Enrique Hernández-Lemus [3,4,*]

[1] *Autonomous University of México City, México*
[2] *Sociomedical Research Unit, National Institute of Cardiology 'Ignacio Chávez', México*
[3] *Computational Genomics Division, National Institute of Genomic Medicine (INMEGEN), México*
[4] *Centro de Ciencias de la Complejidad, Universidad Nacional Autónoma de México, 04510 Mexico City, Mexico*

Correspondence*:
Enrique Hernández-Lemus
ehernandez@inmegen.gob.mx

FUNDAMENTALS OF NETWORK THEORY

Definitions

In this appendix we will introduce some general definitions for concepts in network theory and analysis that may be useful.

Degree, Degree centrality

Degree centrality or simply degree is defined as the number k_i of links incident on a node i (i.e., the number of ties that a node has). The degree measures the flow of information through this node in the network. In the case of a directed network two separate degree centralities are defined, in-degree k_i^{in} and out-degree k_i^{out}. Accordingly, in-degree is a measure of the number of links directed to the node and outdegree is the number of links that such node directs to others. The total number of links L of an undirected network is simply:

$$L = \frac{1}{2} \sum_{i=1}^{N} k_i \tag{1}$$

Average degree

Average degree is simply the mean number of connections of the elements of a given network. It provides a sense of the typical connectivity of the network. It also allows the determination of families of network types via their degree distribution around the mean.

$$\langle k \rangle = \frac{1}{N} \sum_{i=1}^{N} k_i = \frac{2L}{N} \tag{2}$$

Degree distribution

It is the probability distribution of the number of connections per node in a given network. This is an extremely relevant mathematical object since different types of distributions will induce or represent different behaviors of the network.

For a network with N nodes the degree distribution is given by:

$$p_k = \frac{N_k}{N}, \quad \text{subject to} \quad \sum_{k=1}^{\infty} p_k = 1 \tag{3}$$

With N_k, the number of nodes having exactly k links.

From equation 3 it can be noticed that the average degree of a network is given by:

$$\langle k \rangle = \sum_{k=0}^{\infty} k\, p_k \tag{4}$$

Or, more commonly, in real networks with a maximum number of connections k_{max}:

$$\langle k \rangle = \sum_{k=0}^{k_{max}} k\, p_k \tag{5}$$

If the distribution is homogeneous (all nodes are equally connected) there is no relevant node, this is similar to having a random (e.g. normal) distribution in which *most* nodes are similarly connected. In contrast, in *long tail* distributions such as scale-free networks, there is a large difference among the connectivity of the least and most- connected nodes. Such highly-connected nodes, known as *hubs* will be quite relevant for the establishment of the main mathematical properties of the network, hence on their biological features.

Shortest path

A shortest or geodesic path, between two nodes in a network is a trajectory with the minimum number of edges. If the network edges are weighted, it is a path with the minimum sum of edge weights. The length of a geodesic path is called geodesic distance or shortest distance. Geodesic paths are not necessarily unique, but the shortest path distance is well-defined since all shortest paths have the same length. The shortest path is often called the distance between nodes i and j, and is denoted by d_{ij}

Connectedness

In graph and network theory, the concept of connectedness or *topological connectedness* is often used. In topology, a topological space is *connected* if it cannot be formed as the union of two disjoint nonempty open sets. In terms of networks, a network is connected if every pair of nodes in the graph is joined by a path. A network is said to be fully connected if it consists in only one *island*. A network is connected if there is a path connecting every pair of nodes i and j, hence there is a finite d_{ij}. Two nodes are disconnected (thus disconnecting the whole network) if such a path does not exist, in which case we have $d_{ij} = \infty$

Clustering coefficient

In network theory a measure of connectedness is the clustering coefficient that represents the degree to which nodes in a graph are clustered. For a node i with degree k_i the *local clustering coefficient* is given by:

$$C_i = \frac{2\,L_i}{k_i\,(k_i - 1)} \tag{6}$$

Here L_i is the number of links between the k_i neighbors of node i. C_i is normalized and thus can be interpreted as the average probability that two nodes, j and k, connected to node i are in turn directly connected to each other. The local clustering coefficient is thus a measure of link density. A densely connected neighborhood will have a larger clustering coefficient.

The global clustering coefficient is the average local clustering coefficient for all nodes in a network, i.e.:

$$\langle C \rangle = \frac{1}{N}\sum_{i=1}^{N} C_i \tag{7}$$

Random network

A random network is a collection of N nodes in which a link between two nodes exists with probability p. It can be defined in two mostly equivalent ways. N labeled nodes are connected with L randomly placed links, this is usually called a $G(N, L)$ model or Erdös and Rényi or fixed links model. You can also build a random network by having the N nodes connected with a constant probability p, called a $G(N, p)$ or Gilbert model.

The mean degree of a random network is given as:

$$\left\langle k^{random} \right\rangle = \frac{2\,\langle L \rangle}{N} = p(N-1) \tag{8}$$

Notice that equation 8 as compared to equation 4 reveals the effect of the average probability p as representative for the distribution p_k. Careful analysis of most real networks has revealed however, that most of them do not actually follow such random behavior (that has been extremely useful both in the theoretical conceptualization and applications) but they present structural diversity. This fact leads to the proposal of a number of models, the most relevant of which is perhaps the so-called scale free networks.

Scale free networks

The analysis of the empirical degree distribution in a large number of naturally-occurring (as well as in social and technological) networks is, in a good approximation, described by a power-law equation (see Equation 9) in which, the probability of nodes having more and more links become bounded, diminishing depending on the value of the exponent γ.

$$p_k = \alpha k^{-\gamma} \tag{9}$$

The constant α is determined via the normalization condition $\sum_k p_k = 1$, thus:

$$\alpha = \frac{1}{\sum\limits_{k=1}^{\infty} k^{-\gamma}} = \frac{1}{\zeta(\gamma)} \tag{10}$$

Here ζ is Riemann's zeta function. In view of this, a major difference between a random and a scale-free network lies in the high k-value tail of the degree distribution p_k.

Degree correlations

An interesting phenomenon that arises in broad degree distributed networks such as scale-free networks lies in the way highly connected nodes, or hubs are connected in the network. These connectivity patterns induce the so-called degree correlations that in turn led to three different types of behaviors of networks. Networks are *assortative* whenever similarly connected nodes tend to connect with each other (hubs with other hubs and less-connected nodes among themselves). In the contrary case (hubs tend to link to less-connected nodes and viceversa) the networks are called *disassortative*. If none of these behaviors is *evident* (i.e. statistically significant), networks are called *neutral*.

Robustness

Networks are known to play central roles in the development of robustness in biological systems. Cellular robustness for instance is currently understood to be encoded by an intricate tangle of regulatory, signaling and metabolic networks.

It is known, that the structure of the underlying network plays a role in a system's ability to survive attacks on the form of either random failures or specific *insults*. Networks are also involved in the emergence of cascading failures, that constitute a damage mechanism often found in actual biological systems.

Community structure

A network community or *module* is defined as a set of nodes within a network that have a higher likelihood of (intra-)connection to each other than to (inter-) connection with nodes from other communities (see *Modules*).

Spreading phenomena

A relevant application of the concepts of network science in biology is related to flow (of matter, energy or, more generally information) within a network. The mass and energy fluxes on a metabolic network or the spreading of disease in a networked population are both cases of spreading phenomena on a network. The recent rise of the COVID-19 pandemic has without doubt left a lasting impression upon most of us regarding the extraordinary efficiency of the world wide *network of human contacts* (even incidental ones) to act as chains of transmission. The concepts of *critical percolation limit* and *epidemic threshold* have been developed to analyze the phenomenon of information super-spreading on networks.

Component

A network component, often called a connected component or island, in an undirected network is a subgraph (a part of the network) in which any two nodes are connected to each other by one or more paths, and which is connected to no additional nodes in the supergraph (the full network).

Edge Betweenness

or Edge Betweenness Centrality is a centrality measure for the links, it is defined as the number of shortest paths that go through a given link in a network.

Giant Connected Component (GCC)

A giant connected component is a connected component of a given network that contains a significant fraction (more than 50 %) of the nodes of the network.

Module

Network modules (also called Communities) are understood as subnetworks formed by sets of nodes (or vertices) that are more densely connected among themselves than with the rest of the network. Modules are often viewed as semi-autonomous (but not independent) components of a network that are responsible for functionality in real networks.

Network modules play a relevant role in the way we understand specific biological functions as they are encoded in biomolecular networks. The modularity approach tries to analyze how nodes form functional modules associated to similar features.
